# The cGAS–STING pathway in cancer immunotherapy: prognostic value and therapeutic potential

**DOI:** 10.3389/fimmu.2026.1760947

**Published:** 2026-06-02

**Authors:** Faizah Alabi, Sikiru O. Imodoye, Kamoru A. Adedokun, Mohamed A. Eltokhy, Marina Curcic, Ebenezer Okoyeocha, Ishor Thapa, Sunil Tiwari, Ayorinde V. Ogundele, Tolulope E. Ayo, Omoniyi B. Awe, Muh'd Saheed K. Abd-Rouf

**Affiliations:** 1 Department of Immunotherapeutics and Biotechnology, Jerry H Hodge School of Pharmacy, Texas Tech University Health Sciences Center, Abilene, TX, United States; 2 Department of Oncological Sciences, Huntsman Cancer Institute, University of Utah, Salt Lake City, UT, United States; 3 Department of Immunology, Roswell Park Comprehensive Cancer Center, Buffalo, NY, United States; 4 Department of Pharmacology and Toxicology, College of Osteopathic Medicine, Michigan State University, East Lasing, MI, United States; 5 Biodesign Institute, Arizona State University, Tempe, AZ, United States; 6 Departamento de Ciencias Básicas, Facultad de Medicina, Universidad de La Frontera, Temuco, Chile; 7 University of Texas Southwestern Medical Center, Dallas, TX, United States; 8 Department of Biochemistry and Chemistry, University of Toledo, Toledo, TX, United States; 9 Department of Chemistry, New Mexico Highlands University, Las Vegas, NM, United States

**Keywords:** cancer immunotherapy resistance, cGAS-STING, innate-adaptive immunity, tumor immunology, type I interferon (IFN) signaling

## Abstract

Despite major advances in T cell-directed cancer immunotherapy, many patients fail to achieve durable responses, underscoring the need for approaches that mobilize additional arms of the immune system. The cyclic GMP-AMP synthase–stimulator of interferon genes (cGAS–STING) pathway has emerged as a central cytosolic DNA sensor that initiates robust type I interferon signaling and bridges innate and adaptive antitumor immunity. Clinically, heightened cGAS–STING activity correlates with improved survival and enhanced responsiveness to immune checkpoint blockade in subset of tumor types. Preclinical studies have further demonstrated that the pharmacologic or genetic activation of cGAS–STING suppresses tumor growth, promotes dendritic cell maturation, increases effector immune infiltration, and synergizes with PD-1/PD-L1 inhibition. However, the spectrum of tumors that derive the greatest therapeutic benefit from STING activation and the mechanisms underlying pathway silencing or non-responsiveness remain incompletely defined. In this review, we integrate mechanistic, preclinical, and clinical evidence across solid tumors and hematologic malignancies to delineate the roles of cGAS–STING as both a prognostic biomarker and a therapeutic target. Our synthesis highlights the context-dependent nature of cGAS–STING signaling, with therapeutic outcomes shaped by STING pathway integrity, tumor mutational burden, cytosolic DNA load, and the immunologic composition of the tumor microenvironment. Importantly, we examine emerging evidence that excessive, systemic, or chronic STING activation can drive immune exhaustion, tolerogenic myeloid reprogramming, and treatment-limiting toxicity, which are factors likely to contribute to efficacy limitations of the first-generation STING agonists. We further discuss how rational combination strategies, optimized delivery platforms, and the kinetic control of STING activation may overcome these barriers. Collectively, this synthesis provides a conceptual framework to guide the development of next-generation immunotherapies that leverage cGAS–STING signaling while avoiding the immunosuppressive consequences of dysregulated innate immune activation.

## 1 Introduction

Cancer immunotherapy has revolutionized cancer treatment, yet many patients still do not respond or develop resistance (1). One emerging concept to broaden efficacy is engaging the innate immune system to ignite T cell-mediated antitumor responses. Innate cells [such as dendritic cells (DCs), macrophages, and natural killer (NK) cells] orchestrate downstream T-cell priming by sensing danger-associated molecular patterns (DAMPs) and producing type I interferons (IFN-I) and cytokines (2). In this context, the cyclic GMP-AMP synthase (cGAS)–stimulator of interferon genes (STING) pathway has attracted significant interest as a key cytosolic DNA-sensing mechanism that bridges innate and adaptive immunity (3). Building on this pathway, various STING agonists have been developed. Preclinical studies have demonstrated that the direct activation of STING can lead to significant tumor regression and systemic antitumor immune responses in mice (4). This promising evidence prompted early-phase clinical trials of STING agonists, either alone or combined with other therapies (5–8). However, initial clinical outcomes showed that only a subset of patients experienced partial responses (9, 10). It remains unclear which tumor contexts retain functional cGAS–STING signaling, why numerous malignancies actively suppress this pathway through genetic and epigenetic mechanisms, and how acute STING activation promotes antitumor immunity. In contrast, chronic signaling drives immune exhaustion, stromal remodeling, and tumor promotion. Clarifying these context-dependent dynamics is essential for rationalizing therapeutic strategies that exploit or restore STING pathway activity.

This review consolidates the current understanding of the cGAS–STING pathway, emphasizing its mechanisms of activation and the dual cell-intrinsic and cell-extrinsic effects on tumor immunity. We also survey its roles in various cancers and outline recent preclinical and clinical progress with STING agonists. Importantly, we highlight remaining knowledge gaps and propose future research directions to facilitate the clinical application of cGAS–STING targeting.

## 2 Mechanism of cGAS–STING signaling

### 2.1 Canonical signaling

**Figure 1:**
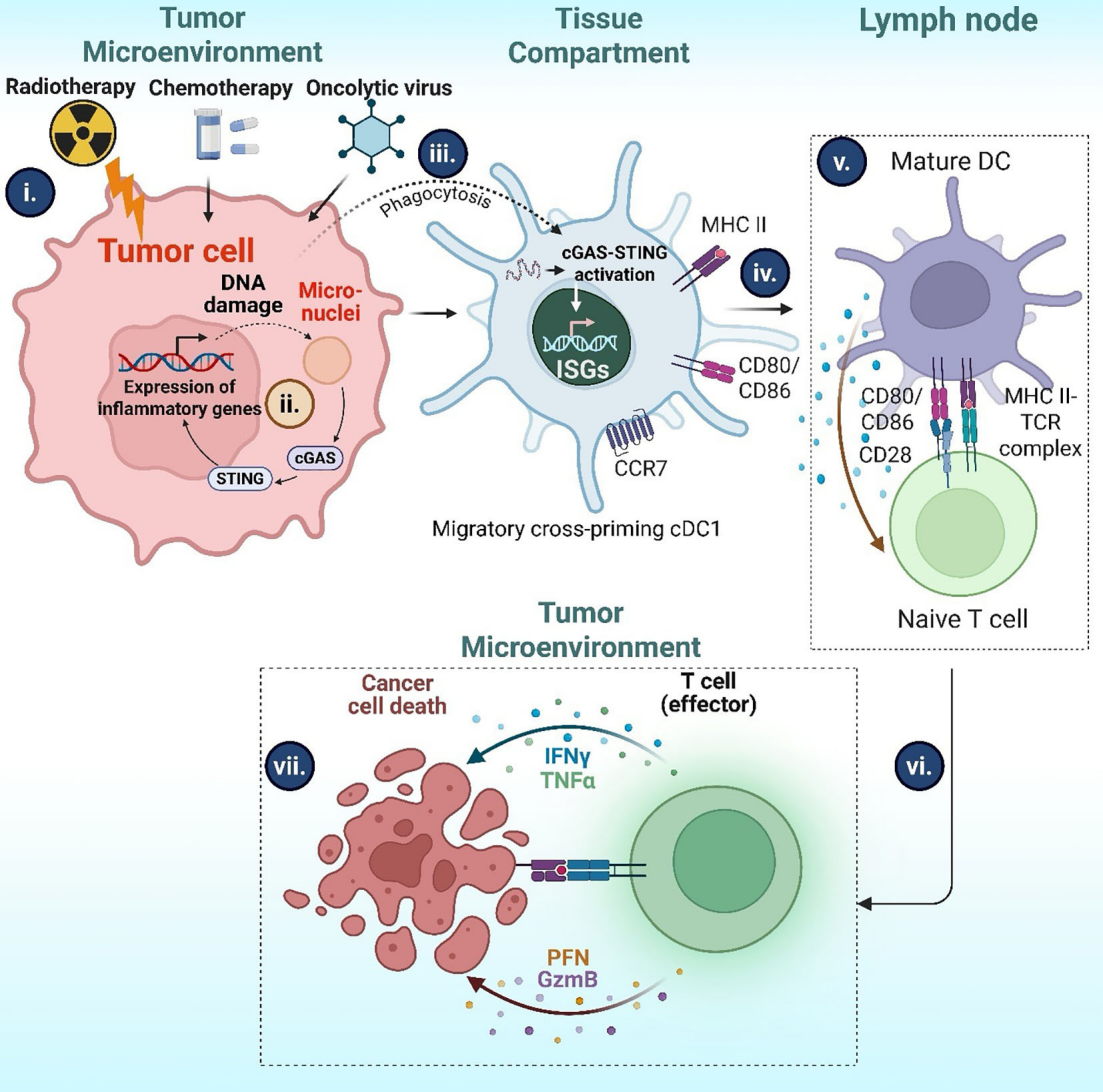
STING-dependent antitumor immunity induced by genotoxic therapy. STING, stimulator of interferon genes. #Genotoxic stress from radiotherapy, chemotherapy, or oncolytic viruses generates cytosolic dsDNA, activating cGAS to produce 2′3′-cGAMP, which binds and activates STING. STING triggers TBK1- and IRF3-dependent induction of type I interferons and NF-κB cytokines. These signals promote dendritic cell (DC) maturation, enhancing antigen uptake and upregulation of MHC-II, CD80/CD86, CD40, and CCR7. CCR7⁺ DCs migrate to draining lymph nodes, where they prime naïve CD8⁺ and CD4⁺ T cells through antigen presentation and costimulatory and cytokine cues. Activated effector T-cells expressing granzyme B, perforin, IFN-γ, and CXCR3 exit the lymph nodes and hone back to the tumor in response to CXCL9/10/11 gradients, where they infiltrate and mediate cytotoxic tumor cell killing. Antigen-bearing tumor cells and mediate cytolysis via granzyme B, perforin, and FasL–Fas pathways, while secreting IFN-γ to amplify local immune activation. The combined cytotoxic and cytokine-driven effects lead to tumor regression and the generation of memory T cells, establishing durable antitumor immunity.

The cGAS–STING signaling pathway is a complex cascade of molecular activations that has emerged as a novel cytosolic DNA-sensing pathway, serving as a critical link between DNA damage and innate immunity (11). It encompasses both the canonical pathway (cGAS-dependent activation of STING) and the non-canonical pathway (cGAS and STING activation independent of each other). In the canonical route, under normal conditions, DNA is confined to the nucleus and mitochondria; its presence in the cytoplasm signals a DAMP, triggering immune responses (12). Mechanistically, the enzyme cGAS detects cytosolic double-stranded DNA in a sequence-independent manner and catalyzes the production of 2′3′-cyclic GMP-AMP (cGAMP), a second messenger that activates the adaptor protein STING located on the endoplasmic reticulum (ER) (13, 14). cGAMP then binds to STING, prompting its translocation to the Golgi apparatus (15). There, STING oligomerizes and recruits TANK-binding kinase 1 (TBK1). Activated TBK1 phosphorylates interferon regulatory factor 3 (IRF3), causing IRF3 to dimerize and move into the nucleus, where it promotes the transcription of type I IFNs (IFN-α/β) and an array of interferon-stimulated genes (ISGs) (15). Concurrently, STING also activates NF-κB via the non-canonical pathway, which induces the production of pro-inflammatory cytokines such as tumor necrosis factor-alpha (TNF-α) and interleukin-6 (16, 17). Collectively, these coordinated events link cytosolic DNA sensing to interferon production and inflammatory signaling, bridging innate and adaptive immunity (**Figure 1**). This dual activation of IRF3 and NF-κB pathways results in a robust inflammatory and antitumor immune response. Given the dominant role of dsDNA in activating the STING pathway, several events are thought to initiate STING signaling. These includes the natural genomic instability of tumor cells, oxidative stress, radiation, and chemotherapy-induced DNA damage and replication, if DNA leaks into the cytoplasm (18, 19). The overall effect is the activation of interferon pathways, which in turn activate both innate and adaptive immune cells. This activation promotes IFN production, dendritic cell maturation, tumor-associated macrophage (TAM) and NK cell activation, and the chemokine-driven recruitment of cytotoxic T lymphocytes (CTLs), collectively enhancing the antitumor immune response. While the central role of cGAS–STING signaling in initiating innate immune responses and antitumor immunity is well established, its activity is tightly regulated to prevent aberrant activation and maintain immune homeostasis.

Contrary to early research, accumulating evidence now indicates that cGAS is not exclusively cytosolic and that the vast majority of resting endogenous cGAS is frequently enriched in the nucleus in both human and mouse cells and tightly tethered to chromatin, a mechanism now recognized to prevent the hyperactivation of self-proteins that could result in autoimmunity (20). Mechanistically, structural and biochemical studies have shown that nucleosomes directly restrain cGAS by binding to cGAS with high affinity via histone surfaces. This configuration occludes productive DNA-binding interfaces required for active cGAS-DNA assembly, thereby suppressing enzymatic activity in the nucleus (21). The disruption of the defined tethering can render cGAS constitutively active, highlighting that nuclear sequestration is not a passive localization phenomenon but a dominant pathway of control (20, 22). It should be noted that this updated model complements rather than replaces the canonical cytosolic mechanism and helps explain why productive cGAS–STING signaling in cancer is particularly linked to contexts that generate extranuclear DNA (micronuclei or cytosolic DNA) or otherwise relieve chromatin-imposed inhibition.

### 2.2 Non-canonical signaling

Rather than transient cytosolic DNA exposure, non-canonical STING signaling in cancer is initiated by persistent endogenous DNA stress, including oncogene-driven replication stress, chromosomal instability (CIN), micronucleus formation and rupture, and exposure to genotoxic agents such as etoposide (23–25). These chronic insults generate sustained DNA damage signals that bias STING activation toward inflammatory and stress-adaptive outputs rather than interferon-dominant immunity. At the molecular level, nuclear DNA damage is first detected by the ataxia telangiectasia mutated–poly(ADP-ribose) polymerase 1 (ATM–PARP1) signaling axis. This results in ATM-dependent phosphorylation of p53 at Ser15 and PARP1-mediated chromatin signaling without inducing overt cell death. The nuclear DNA sensor interferon-inducible protein 16 (IFI16), which constitutively associates with damaged chromatin, binds phosphorylated p53 and shuttles this complex to the cytosol, where it assembles on ER-resident STING. Importantly, this mode of STING engagement does not induce the conformational activation, oligomerization, or Golgi trafficking that typifies canonical cGAS–STING signaling. Instead, IFI16 functions as a molecular adaptor that enables the damage-specific recruitment of TRAF6 to STING at the ER membrane. TRAF6 catalyzes K63-linked polyubiquitination of STING, creating a signaling scaffold that recruits TAK1-binding protein 2/3 (TAB2/3) and activates TAK1, leading to phosphorylation of the IKK complex. From this point, non-canonical STING signaling diverges into two closely related but mechanistically distinct inflammatory outputs, which frequently coexist in tumors.

First, the non-canonical STING pathway preferentially induces NF-κB-dependent inflammatory gene programs while eliciting only limited and delayed IFN-β expression. Transcriptionally, this NF-κB-biased signaling induces cytokines such as interleukin-6 (IL-6) and CCL20. IL-6 secretion establishes a pro-survival, stress-adaptive inflammatory state, whereas CCL20 promotes chemotactic and tissue-remodeling programs that are not typical of canonical STING responses (23). Importantly, pharmacologic or genetic inhibition of ATM, PARP1, IFI16, p53, TRAF6, or Ubc13 selectively abrogates NF-κB activation and downstream cytokine induction following nuclear DNA damage. These findings suggest that NF-κB-biased STING signaling triggered by nuclear DNA damage depends on a distinct damage-sensing and ubiquitin-signaling axis rather than the canonical cytosolic DNA pathway. In this context, STING operates as a nuclear damage-responsive inflammatory hub that preferentially drives pro-survival and tissue-remodeling cytokine programs.

Second, NF-κB-independent inflammatory outputs can also emerge downstream of non-canonical STING activation in a TBK1- and IRF3-independent manner. The non-canonical NF-κB responses trigger p52–RELB nuclear translocation and limit type I interferons and canonical NF-κB (26). This mechanism emerges as a critical negative regulator of STING effector mechanisms, with important biological consequences for cancer immune escape and metastasis. Using human metastatic datasets and genetically controlled breast and lung cancer models, Bakhoum et al. showed that cell-intrinsic chromosomal instability drives persistent chromosome missegregation. This leads to micronucleus formation and rupture, releasing genomic DNA into the cytosol and chronically activating STING signaling in a TBK1-independent manner. Rather than inducing type I interferon, this pathway preferentially engages non-canonical NF-κB signaling through RELB/p52. The resulting transcriptional program promotes inflammatory signaling, epithelial–mesenchymal transition (EMT), invasive behavior, and metastatic colonization. Suppressing chromosomal instability, micronuclear rupture, STING, or non-canonical NF-κB components selectively impairs metastasis, establishing CIN-driven non-canonical cGAS–STING signaling as a tumor-intrinsic driver of metastatic progression (19).

Beyond tumor cell-intrinsic effects, non-canonical STING signaling exerts potent immunomodulatory consequences within the tumor microenvironment (TME). In a colon cancer model, uptake of tumor-derived DNA by DCs activates STING and induces TBK1-mediated phosphorylation of NF-κB2 (p100), promoting its processing into p52 and nuclear translocation of RelB–p52 complexes (26). This non-canonical NF-κB activation actively suppresses antitumor immunity by inhibiting canonical NF-κB (RelA) binding to the IFN-β promoter, thereby attenuating type I interferon production despite ongoing STING engagement. As a result, DC maturation, CD8^+^ T-cell priming, and effector responses are restrained. Consistent with this mechanism, genetic or pharmacologic inhibition of non-canonical NF-κB signaling in DCs restores type I interferon production, enhances DC maturation, reinvigorates CD8^+^ T-cell responses, and improves tumor control *in vivo*.

Collectively, these studies have established a unified model in which chronic DNA damage-driven, non-canonical STING signaling promotes tumor progression through two interconnected routes: i) NF-κB-dependent and independent inflammatory programs that enhance tumor cell survival, plasticity, and metastasis; and ii) active suppression of protective type I interferon-mediated antitumor immunity within the TME. In this context, STING functions not as a guardian of immune surveillance but as a maladapted inflammatory sensor that tumors exploit to support progression and immune evasion.

## 3 Downstream activities of cGAS–STING signaling: cell-intrinsic and cell-extrinsic roles

Once activated, the cGAS–STING pathway can elicit a variety of downstream effects on tumor cells and the tumor immune microenvironment (TiME). Notably, the cGAS–STING signaling pathway plays a dual role in cancer: it can act as a tumor-suppressive mechanism (especially via acute activation, leading to immune clearance of cancer cells), but chronic or context-specific activation can promote tumor progression. Subsequently, we discuss the cell-intrinsic effects of cGAS–STING within tumor cells and the cell-extrinsic effects on immune cells in the TiME, highlighting how the pathway can both inhibit and inadvertently support tumor growth.

### 3.1 Cell-intrinsic effect: tumor-suppressive and tumor-promoting mechanisms

Within tumor cells, cGAS–STING signaling influences cell fate and exerts potent tumor-suppressive effects. The acute activation of cGAS–STING can induce oncosis—a state of tumor cell cycle arrest or cell death (27). For example, cGAS–STING can trigger senescence in cancer cells, accompanied by a senescence-associated secretory phenotype (SASP) (28). The SASP involves the secretion of chemokines, IFNs, and proteases that reinforce growth arrest and attract immune cells to eliminate senescent tumor cells (29, 30). In some contexts, STING activation can also directly induce apoptosis of tumor cells (31, 32). These cell-intrinsic antiproliferative effects function as a tumor-suppressor mechanism, in which damaged or genomically unstable cells that accumulate cytosolic DNA may be forced into senescence or death via STING, thereby preventing the formation of premalignant lesions or malignant progression. Indeed, the loss of STING in tumor cells has been associated with unchecked tumor growth and worse patient outcomes in certain cancers, emphasizing its intrinsic tumor-suppressor role (33–38).

Paradoxically, persistent or chronic cGAS–STING signaling in tumor cells, particularly those with high chromosomal instability, promotes cancer stemness, enhances tumor cell survival, and drives metastatic potential (19, 39). Several mechanisms have been proposed to explain these tumor-promoting activities, including the activation of SASP, autophagy induction, and non-canonical NF‐κB (40). In breast and lung cancer models, chronic STING stimulation was shown to activate non-canonical NF-κB activity, which mediates metastasis in a tumor cell-autonomous fashion (19). This oncogenic role is further supported by functional experiments showing that depletion of STING, RELB, or p100 (encoded by NF-κB2) in CIN-high cells reduces metastatic dissemination, improves overall survival, and decreases both *in vitro* and *in vivo* invasiveness. These results align with previously reported roles of the non-canonical NF-κB pathway in EMT, tumor cell invasion, and metastasis (41).

Emerging evidence indicates that cGAS–STING signaling is a key driver of oncogene-associated cancer progression. Elevated cGAS–STING signaling in breast tumors correlates with higher tumor grades (42), suggesting a link to more aggressive disease. At the molecular level, cGAS–STING activation correlates with elevated expression of replication stress-inducing oncogenes, such as Cyclin E1 and *c-Myc* (42). Downstream inflammatory mediators, particularly pSTAT1 and pTBK1, correlate with Cyclin E1 and c-Myc expression, suggesting that STING-mediated signaling may modulate oncogene expression in tumor cells, thereby selecting for more aggressive clones. Furthermore, analyses of The Cancer Genome Atlas (TCGA) cohort have revealed that higher cGAS–STING scores are linked to increased mRNA levels and genomic gains of replication stress-related oncogenes, underscoring a potential cell-autonomous pathway through which cGAS–STING promotes tumor progression. Collectively, these observations highlight a mechanism whereby intrinsic cGAS–STING activity fosters oncogene-driven replication stress, contributing directly to tumorigenesis in breast cancer.

### 3.2 Cell-extrinsic effects: regulation of immune cells

Beyond tumor-intrinsic effects, the cGAS–STING pathway plays a crucial role in the TiME by linking innate and adaptive immunity. In immune cells, cGAS–STING activation can lead to the production of pro-inflammatory cytokines (mainly type I IFNs) and the chemokine-driven recruitment of lymphocytes, which are central to antitumor immunity. However, cGAS–STING-driven inflammation in the TME can also have unintended consequences, such as the recruitment of immunosuppressive cells or the formation of physical barriers to immune infiltration. In this section, we highlight the mechanisms driving the dual role of cGAS–STING in the TME, with a special focus on neutrophils and neutrophil extracellular traps (NETs) as key mediators of the innate immune arm.

#### 3.2.1 Neutrophils as first responders in cGAS–STING signaling

**Figure 2:**
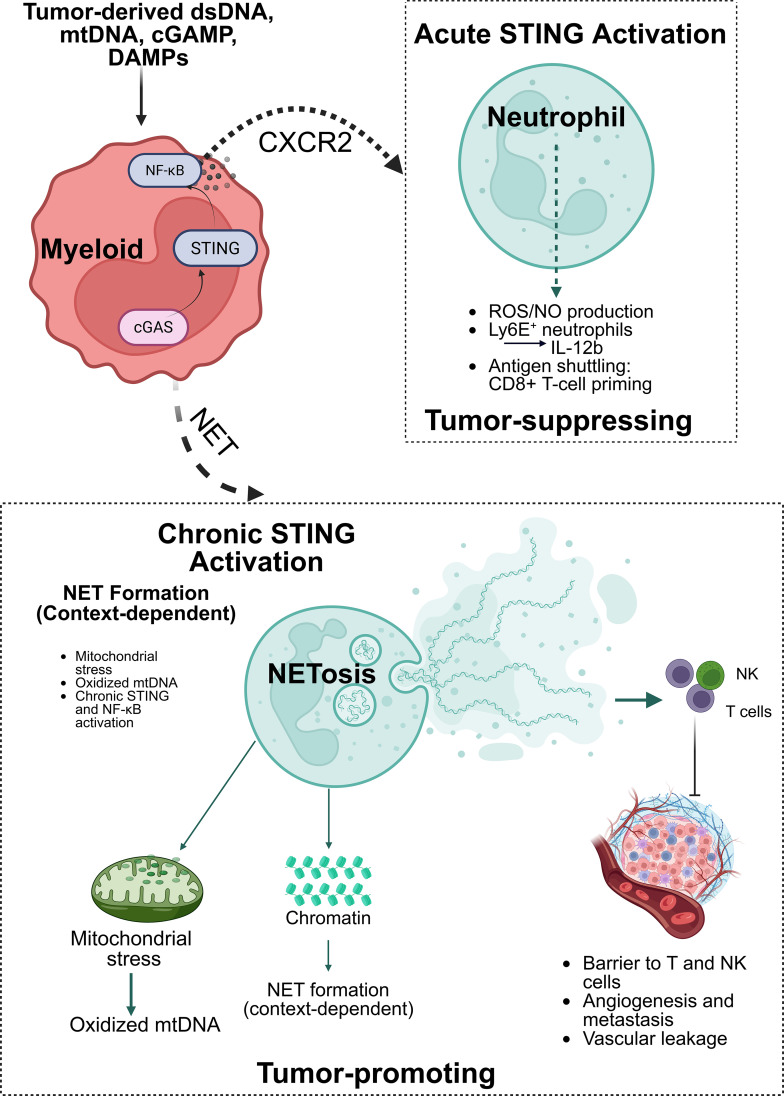
Stepwise mechanism of cGAS–STING pathway activation by neutrophils. cGAS, cyclic GMP-AMP synthase; STING, stimulator of interferon genes. Tumor-derived dsDNA, mtDNA, cGAMP, and other DAMPs activate cGAS-STING in host myeloid cells, leading to chemokine production that drives CXCR2+ neutrophil recruitment into the tumor. Type I IFN exposure subsequently activates neutrophils to enhance antitumor effector functions such as reactive-oxygen species- nitric oxide (ROS/NO) generation, emergence of Ly6E+ neutrophils, and antigen shuttling that primes CD8+ T-cells. Under conditions such as mitochondrial stress, oxidized mtDNA release, or chronic STING-NF-κB activation, neutrophils undergo NETosis and NETs release, which exert dual roles; acute activation reinforces innate immune signaling through secondary DNA sensing, whereas chronic activation creates physical barriers that impede CD8+ T-cells and NK cell infiltration, promote angiogenesis, metastasis, and induce vascular leakage. Together, these processes illustrate the context-dependent shift of neutrophils from STING-amplifying antitumor effectors to NET-driven facilitators of tumor progression.

Neutrophils act as early responders within a broader cGAS–STING-driven innate immune cascade involving multiple immune cell populations (**Figure 2**). While much early work on tumor STING signaling has focused on dendritic cells and macrophages, recent evidence identifies neutrophils as important first responders to STING activation in tumors. Studies using conditional knockout mice have demonstrated that deletion of cGAS or STING in myeloid cells, including neutrophils, abolishes the tumor’s responsiveness to STING agonists. This finding established that host myeloid DNA sensing, rather than tumor-intrinsic STING activity, is essential for generating IFN-driven chemokine responses, lymphocyte recruitment, and CD8^+^ T-cell priming (43). Thus, neutrophils actively contribute to the sequence linking cytosolic DNA sensing with adaptive immune activation, rather than serving as passive responders. 

Mechanistically, the intratumoral delivery of the STING ligand (cGAMP) induced NF-κB-dependent expression of C-X-C Motif Chemokine Ligand 1 (CXCL1) and CXCL2, driving C-X-C Motif Chemokine Receptor 2 (CXCR2)-mediated neutrophil recruitment to tumors. Blocking CXCR2 suppressed neutrophil infiltration and diminished the antitumor efficacy of cGAMP, confirming that neutrophils amplify STING-driven immunity (44). Once recruited, neutrophils exposed to type I IFNs, such as IFN-β, displayed enhanced cytotoxicity by producing reactive oxygen species, and STING activation failed in mice lacking IFN receptors, confirming the central role of type I IFN signaling. Moreover, neutrophils appear to help bridge innate and adaptive immunity, as IFN-primed neutrophils supported CD8^+^ T-cell training in draining lymph nodes, possibly by serving as antigen carriers or influencing DC–T-cell interactions.

Adding to this picture, Benguigui et al. identified a subset of neutrophil (Ly6E^+^ neutrophils) that emerges upon STING activation. These cells secreted IL-12b, activating T cells and correlating strongly with responses to checkpoint blockade therapy in both mice and patients (45). Interestingly, these neutrophils only appeared when tumor cells exhibited active STING signaling, underscoring that the cGAS–STING pathway tightly regulates neutrophil phenotype and function. Clinical data reinforce these findings: in hepatocellular carcinoma (HCC) patient datasets, high cGAS–STING activity correlates with immune infiltration, including neutrophils, and with expression of pathway genes, which predict better patient outcomes (46). Meanwhile, neutrophils can also themselves be a source of cytosolic DNA that activates STING. In conditions of cellular stress, neutrophils may release mitochondrial DNA (mtDNA) into the cytosol. Both Liu et al. and Pan et al. showed that oxidized mtDNA leaking from damaged mitochondria in neutrophils can trigger cGAS–STING within those neutrophils. In high-fat diet-induced HCC, this led to gasdermin D-mediated neutrophil pyroptosis and the activation of NLRP3 inflammasome, fueling a highly inflammatory microenvironment that promoted tumor progression (47, 48).

Similarly, in triple-negative breast cancer (TNBC), inhibition of carnitine palmitoyltransferase 1A (CPT1A), a key fatty acid oxidation enzyme, induced mitochondrial stress and mtDNA release, thereby activating cGAS–STING in tumor cells. This led to IFN-stimulated neutrophil recruitment via chemokines such as CXCL10. These neutrophils exhibited tumoricidal phenotypes, producing reactive oxygen species (ROS) and nitric oxide (NO). Depletion of neutrophils, but not CD8^+^ T cells, abolished the therapeutic benefit, establishing them as primary mediators of tumor control. Single-cell RNA-seq further confirmed the enrichment of NETosis-related gene programs, linking cGAS–STING signaling to NET-associated antitumor functions, while *in vivo* pharmacologic inhibition of CPT1A using teglicar highlighted CPT1A inhibition as a potent pathway to target neutrophils in cGAS–STING signaling (49).

In summary, neutrophils are now recognized as active participants (not just bystanders) in STING-driven antitumor immunity. STING activation in the TME mobilizes neutrophils, alters their phenotype toward an antitumor state (at least transiently), and utilizes their effector functions to amplify immune responses. However, as discussed next, neutrophils can also form NETs that may have pro-tumor consequences in the context of chronic STING activation.

##### 3.2.1.1 Neutrophil extracellular traps as mediators of cGAS–STING signaling

A striking intersection between neutrophils and cGAS–STING signaling is the formation of NETs. NETs are web-like structures composed of extruded chromatin (DNA decorated with citrullinated histones) and granular enzymes. Neutrophils deploy NETs to trap and kill microbes, but NETs can also profoundly influence the TME. Depending on the context, NETs can enhance antitumor immunity or promote tumor progression.

##### 3.2.1.2 Mitochondrial stress and NET formation

In HCC models driven by a high-fat diet, Pan et al. demonstrated that metabolic and mitochondrial stress in neutrophils triggers NET release through a STING-dependent mechanism. Oxidative damage leads to mtDNA leakage in neutrophils, which activates their internal cGAS–STING and NF-κB pathways (48), causing gasdermin D-mediated membrane poration and NLRP3 inflammasome activation, essentially a programmed cell death that expels NETs. The expelled NETs rich in DNA and proteases can deposit in tumors and form a barrier that physically excludes cytotoxic lymphocytes (T and NK cells) from infiltrating tumor tissue. This results in an immune-excluded tumor phenotype, in which immune cells are trapped in stromal areas and cannot effectively contact cancer cells. Indeed, in the Pan et al. study, degrading NETs with DNase I or reducing neutrophil mitochondrial stress with a PPAR-agonist restored lymphocyte infiltration and significantly reduced tumor burden. These findings revealed a paradoxical pro-tumor axis: neutrophils responding to chronic, metabolic stress-driven STING activation can undergo NETosis, and the resulting NETs shield the tumor from immune attack (48).

##### 3.2.1.3 Amplification loops and intercellular signaling

NETs are not inert debris; the extracellular DNA they contain can itself activate cGAS in adjacent immune or stromal cells, creating a feed-forward loop of DNA sensing and inflammation. Reviews by Adrover et al. and Liu et al. emphasized that NET-derived DNA, particularly mtDNA, mimics bacterial DNA, stimulating strong innate responses and chronic inflammatory signaling. In the tumor context, this cycle of mtDNA release, cGAS–STING activation, and NET formation can perpetuate inflammatory damage, tissue remodeling, and immune dysfunction, thereby facilitating tumor growth and metastasis (47, 50).

##### 3.2.1.4 Dual roles in tumor immunity

While NETs can occasionally constrain tumors by trapping circulating malignant cells or restricting localized infection, a growing body of evidence supports their pro-tumor functions (51). NETs promote angiogenesis, facilitate extravasation, and remodel the extracellular matrix to facilitate tumor cell migration. In both liver and pancreatic cancers, NETs have been shown to enhance tumor invasion and vascular penetration, reinforcing the concept that NETs often act as scaffolds of metastasis rather than barriers to it (52–54).

Tumor-derived dsDNA, mtDNA, cGAMP, and other DAMPs activate cGAS–STING in host myeloid cells, leading to chemokine production that drives CXCR2+ neutrophil recruitment into the tumor. Type I IFN exposure subsequently activates neutrophils to enhance antitumor effector functions such as ROS/NO generation, emergence of Ly6E^+^ neutrophils, and antigen shuttling that primes CD8^+^ T cells. Under conditions such as mitochondrial stress, oxidized mtDNA release, or chronic STING–NF-κB activation, neutrophils undergo NETosis and NET release, which exert dual roles; acute activation reinforces innate immune signaling through secondary DNA sensing, whereas chronic activation creates physical barriers that impede CD8^+^ T cells and NK cell infiltration, promote angiogenesis and metastasis, and induce vascular leakage. Together, these processes illustrate the context-dependent shift of neutrophils from STING-amplifying antitumor effectors to NET-driven facilitators of tumor progression.

#### 3.2.2 cGAS–STING signaling influences macrophage plasticity

Macrophage polarization is closely related to the activation of the cGAS–STING pathway. The activation of STING stimulates the expression of IFN-I and pro-inflammatory cytokine production through TBK1, IRF3, and NF-κB, which can directly reprogram TAMs toward the M1-like phenotype. A study by Gomes et al. showed that in *Brucella abortus* infection, the STING activation results in M1 polarization by stabilizing hypoxia-inducible factor-1α (HIF-1α). As a result, energy metabolism shifts toward glycolysis, IL-1β and nitric oxide production increase, and mitochondrial reactive oxygen species production increase, supporting an M1-like inflammatory profile (55, 56). Moreover, M1 macrophage stimulation occurs through the mtDNA–mechanistic target of rapamycin complex 1 (mTORC1) pathway.

In cancer, the role of the cGAS–STING pathway on macrophage polarization is complex and double-edged. In the TME, acute STING activation or the use of STING agonists was associated with increased M1 polarization and, consequently, enhanced cytotoxic T-cell recruitment and antitumor effect (57). Moreover, when using STING agonists in a colorectal cancer mouse model, the results showed increased antitumor activity through targeting tumor-associated macrophages (58). STING were employed to overcome PARP resistance; in a BRCA1-deficient model, they stimulated M1 polarization of macrophages by reprogramming tumor-associated macrophages (59). Additionally, the use of STING agonists in hormone-dependent solid tumors, alongside endocrine therapies, was shown to improve antitumor efficacy (60). In gastric cancer, STING activation produced similar effects, enhancing antitumor activity by inducing apoptosis of gastric cancer cells. The proposed mechanism involves STING-induced IL6R–JAK–IL24 pathway activation in macrophages (61). STING activation has also been linked to reduced liver metastasis in colorectal cancer, likely due to IRG1-induced transcription factor EB (TFEB) nuclear translocation (62).

#### 3.2.3 cGAS–STING signaling enhances antigen presentation on dendritic cells

The extrinsic effect of cGAS–STING on cells is also well documented in host antigen-presenting cells, especially in DCs that are key for initiating adaptive immunity. Foundational studies have shown that STING in host DCs is essential for spontaneous T-cell priming against tumors and for radiotherapy-induced antitumor immunity (63). Tumor-derived DNA or cGAMP can be released from tumor cells via autophagy-linked secretion or gap junctions and enter host DCs or macrophages, triggering STING signaling in those cells (64). This causes a local increase in IFN-I, which activates conventional DCs expressing Basic Leucine Zipper ATF-Like Transcription Factor 3 (BATF3+) to cross-prime CD8^+^ T cells against tumor antigens (65). Additionally, the secretion of IFNs and cytokines can upregulate costimulatory molecules like CD80, CD86, and CD40 on the DCs, further boosting the DC’s ability to process antigens and prime the T cells effectively. The pathway can also contribute to reducing immunosuppressive cells, such as regulatory T cells (66).

In melanoma, evidence indicates that STING signaling facilitates the induction and formation of tertiary lymphoid structures (TLSs) within the TME (67). In a murine model of colitis-associated cancer, STING-deficient mice exhibited a higher incidence of tumor development, suggesting an antitumor role for the STING pathway (68). Similarly, in bladder cancer, patients who responded to Bacillus Calmette-Guérin (BCG) therapy displayed increased STING expression and immune cell activation (69). In summary, the activation of the cGAS–STING pathway appears to be essential for DC-mediated tumor regression, particularly in therapeutic contexts such as radiation therapy and immune checkpoint inhibitors (ICIs), which robustly stimulate STING signaling to promote tumor-infiltrating CD8^+^ T-cell responses.

#### 3.2.4 cGAS–STING pathway activates natural killer cells

STING activation also plays a critical role in modulating the TME by recruiting NK cells and promoting an inflamed milieu conducive to antitumor immune responses. The activation of STING facilitates the proliferation and expansion of specific NK cell subsets, such as TCF1-positive NK cells, thereby enhancing innate immune surveillance. Furthermore, STING signaling synergizes with macrophages to induce the release of pro-inflammatory cytokines, including interleukin-18 (IL-18), interleukin-1 beta (IL-1β), and 4-1BB ligand (4-1BBL). These cytokines are instrumental in orchestrating a durable antitumor immune response. Empirical evidence from various cancer models, including melanoma, colorectal, and pancreatic carcinomas, underscores the pivotal role of STING pathway activation in mediating NK cell-dependent tumor rejection, inhibiting hepatic metastasis of colorectal malignancies, and potentiating the efficacy of STING-based cancer vaccines (62, 70, 71).

#### 3.2.5 cGAS–STING regulates B and T lymphocytes

The activation of STING enhances B-cell receptor signaling *in vitro*. Moreover, STING activation augmented B-cell receptor-induced NF-κB responses, highlighting B cells’ ability to integrate innate and adaptive immunity via the STING pathway (72). A study by Tang et al. showed that STING agonists induced apoptosis in malignant B cells, suggesting that STING activation may be essential for B-cell survival (32). However, a study by Green et al. showed that deficiencies in IFN-I or cGAS in B cells did not affect their physiological germinal center response. In contrast, a deficiency in TLR7 diminished B-cell germinal center participation, highlighting that, *in vivo*, B-cell receptor–cGAS activation is not necessary for germinal center participation (73).

Furthermore, in human B cells, it was shown that IFN-I production is not increased in response to cytoplasmic DNA, suggesting that the cGAS–STING pathway is regulated differently in B cells (74). All of these lines of evidence show that the *in vitro* stimulation of STING is directly related to B-cell receptors, while *in vivo* studies of the relationship between STING and B cells are uncertain. Moreover, the simultaneous activation of the lymphotoxin-β receptor (LTβR) and the STING pathway in tumors induces germinal center-like B-cell responses, thereby enhancing CD8^+^ T cell-dependent tumor suppression (75). However, the chronic activation of the STING pathway expands IL-35^+^ regulatory B cells (B-regs), suppressing T cells, highlighting the cGAS–STING pathway’s effects on both B cells and their subset, regulatory B cells (76).

The chronic stimulation of the cGAS–STING pathway triggers apoptotic pathways via IRF3, leading to cell death, reduced T-cell function, and proliferation. This effect is independent of IFN-I (77). In adoptive cell therapy, STING activation was associated with improved T-cell trafficking and reduced levels of immunosuppressive cells, while low-dose activation was associated with enhanced IFN-I production and cytolytic activity. However, chronic STING activation led to exhaustion (4, 78).

Furthermore, the STING pathway was shown to stimulate regulatory T cell (Treg) differentiation by cooperating with IL-2 receptor (IL-2R) and transforming growth factor-β (TGF-β) in tumor models (77). The sensing of DNA by cGAS–STING was shown to aid in maintaining a stem-like memory in CD8^+^ T cells (79). All of this highlights the double-edged nature of cGAS–STING pathway activation in T-cell regulation.

Additionally, STING can activate T cells indirectly via IFN-I, a model similar to many vaccines in activating T-cell responses (80). Another mechanism is activating the cGAS–STING pathway in antigen presenting cells (APCs) to generate chemokines such as CXCL9 and CXCL10, which activate T cells in tissues. Moreover, chemokines stimulate IFN-I release, which promotes T-cell differentiation (81). The coactivation of STING and LTβR stimulates TLS formation, creating a permissive environment for APCs and, consequently, T-cell activation (75).

#### 3.2.6 cGAS–STING drives immunosuppressive remodeling of the tumor microenvironment

Tumors exploit cGAS–STING-mediated inflammatory signaling to reshape the TME through various mechanisms. Chronic cGAS–STING signaling can induce immunosuppressive cytokines such as IL-10 and TGF-β, which impair CD8^+^ T and NK cell function and foster Treg accumulation (82). A recent report identified IL-35 as a potent cytokine produced by regulatory B cells in response to STING stimulation (76). This cytokine suppresses NK cell activity and impairs NK cell-mediated cytotoxicity in the pancreatic ductal adenocarcinoma (PDAC) *in vivo* model. Interestingly, blocking IL-35 in B-regs significantly improves tumor control, highlighting the STING–IRF3–IL-35 axis as a key driver of immunosuppressive reprogramming and potential therapeutic target (76). Additionally, the continuous production of specific cytokines, for example, chronic STING activity, increases IL-6 secretion, which can support tumor cell survival via STAT3 signaling pathways, as observed in chromosomally unstable triple-negative breast cancers (83).

Beyond cytokine signaling, chronic inflammation downstream of cGAS–STING reprograms stromal and myeloid compartments to favor tumor progression. Tumor-associated macrophages are skewed toward an M2-like phenotype that supports tumor growth (25). At the same time, fibroblast recruitment enhances extracellular matrix deposition and fibrosis, forming a physical barrier that restricts immune infiltration (84). Moreover, the cGAS–STING pathway enhances the recruitment of myeloid-derived suppressor cells (MDSCs), supporting their immunosuppressive activity in a murine model of lung carcinoma (85). Also, in mouse models of colon adenocarcinoma, STING orchestrates the mobilization and reprogramming of monocytic myeloid-derived suppressor cells (M-MDSCs) after radiation exposure (86). Through innate immune sensing of cytosolic DNA, IRF activates STING, triggering IFN-I signaling that induces CCL2, CCL7, and CCL12 expression in tumor and host cells, thereby recruiting CCR2^+^Ly6C^hi^ M-MDSCs. These immunosuppressive MDSCs limit irradiation efficacy and contribute to tumor relapse. Combined M-MDSC depletion by anti-CCR2 antibodies, STING agonism, and IR synergistically enhances antitumor immunity by augmenting CD8^+^ T-cell activity and increasing CD8^+^/Treg ratios, overcoming radioresistance and leading to durable tumor regression (86). Thus, STING-mediated M-MDSC recruitment represents an immunosuppressive adaptation influencing tumor progression post-IR.

STING also intersects with metabolic immunosuppression through the induction of indoleamine 2,3-dioxygenase (IDO), an enzyme whose metabolite kynurenine suppresses effector T-cell function and facilitates immune escape (87). Interestingly, STING activation can promote tumor growth by inducing IDO expression, as observed in the weakly antigenic LLC model (88). Accordingly, the combination of STING agonists and IDO inhibitors is a potentially effective option for cancer treatment. Meanwhile, tumor antigenicity may be a critical determinant of whether STING signaling induces IDO, fostering immune suppression, or promotes effective antitumor responses.

Another immune escape mechanism linked to chronic cGAS–STING activation is PD-L1 upregulation, leading to T-cell exhaustion and impaired antitumor immunity (89). Although the precise mechanisms remain under investigation, current evidence implicates NF-κB signaling (90). Another potential mechanism may involve IL-6, which serves as a pivotal bidirectional regulatory hub within the cGAS–STING pathway, where it is both induced by STING activation and reciprocally modulates its signaling. In triple-negative breast cancer, STING-mediated NF-κB activation elevates IL-6 and pSTAT3, promoting immune evasion through PD-L1 upregulation (91).

#### 3.2.7 Defining and modeling acute vs chronic STING activation in preclinical studies

Most experimental models of chronic cGAS–STING signaling rely on cell-intrinsic mechanisms that sustain pathway activity. One such model recapitulates tumor-promoting cGAS–STING signaling through tumor-intrinsic stabilization of STING by Galectin-1. Galectin-1 physically interacts with STING and prevents its autophago-lysosomal degradation, prolonging STING protein half-life and maintaining downstream signaling with a pronounced NF-κB bias. This persistent NF-κB-dominant activity drives continuous expression of MDSC-recruiting chemokines, leading to systemic MDSC expansion, lung premetastatic niche remodeling, and enhanced metastatic progression, independent of adaptive lymphocytes (92). Blocking galectin-1-mediated activation blocks this inflammatory circuit and suppresses metastasis without significantly affecting primary tumor growth.

A complementary *in vivo* model is provided by long-term topical exposure to the DNA adduct-forming carcinogen DMBA. Repeated low-dose DMBA treatment over 18–20 weeks induces persistent cytosolic DNA leakage and cGAS-dependent STING activation, characterized by sustained ER exit, phosphorylation, and prolonged nuclear localization of NF-κB and IRF3. This chronic signaling drives continuous production of pro-tumorigenic inflammatory mediators, including IL-6 and CCL5, establishing a persistent inflammatory microenvironment that promotes epithelial tumor initiation and progression. STING deficiency protects against DMBA-induced squamous cell carcinoma, implicating long-term STING activity as a driver of DMBA-induced tumor progression (93). Operationally, chronic cGAS–STING activation is defined by sustained pathway engagement over extended periods, marked by prolonged STING stabilization, persistent ER exit, and continuous NF-κB-dominant transcriptional output. This contrasts with acute activation, which is short-lived, typically induced by exogenous agonists, dominated by IRF3-dependent type I interferon responses, and terminated through STING degradation. Taken together, these signalosomes establish a self-limiting immunosuppressive loop that attenuates antitumor immunity.

## 4 Regulation of cGAS–STING signaling via genetic and epigenetic mechanisms

The cGAS–STING signaling cascade is regulated at multiple levels, including genetic, epigenetic, transcriptional, and post-translational modifications, and modulation by cellular metabolites and ions, among others. In the following sections, we highlight the key modes of cGAS–STING regulation, highlighting how each influences pathway activation, immune outcomes, and potential therapeutic strategies. In human cancers, direct mutations in the MB21D1 (encoding cGAS) and TMEM173 (encoding STING) genes are rare, generally occurring in less than 1% of tumor types. These findings indicate that most cancers preserve the cGAS–STING pathway at the genomic level, and immune evasion likely occurs through epigenetic, transcriptional, post-transcriptional, or post-translational modifications. Conversely, gain-of-function (GOF) mutations, such as V147L, V155M, N154S, and R281Q, in TMEM173 cause constitutive activation, resulting in excessive IFN-I responses (94). This excessive STING activation drives systemic inflammation, vasculopathy, and immune dysregulation and, in extreme cases, severe autoinflammatory diseases such as STING-associated vasculopathy with onset in infancy (SAVI).

### 4.1 Epigenetic regulation

Epigenetic mechanisms critically regulate the expression and activity of cGAS and STING, thereby modulating innate immune sensing and antitumor immunity (95).

#### 4.1.1 DNA methylation

DNA methyltransferases (DNMTs) catalyze the addition of methyl groups to cytosine residues in CpG islands, often resulting in transcriptional silencing (96). Analysis of large-scale datasets, such as TCGA, provides population-level evidence for epigenetic regulation of cGAS. Specifically, TCGA revealed a negative correlation between cGAS, STING, TBK1, and IRF3 gene expression and DNA methylation levels in a cancer type-specific manner (97), suggesting that hypermethylation acts as a suppressive mechanism that silences cGAS across diverse tumor types. Specifically, the promoter of MB21D1 is significantly hypermethylated in melanoma, colorectal carcinoma, liver hepatocellular carcinoma, lung squamous carcinoma, and prostate adenocarcinoma (97, 98). Similarly, TMEM173 shows pronounced promoter hypermethylation in melanoma, breast cancer, lung adenocarcinoma, lung squamous carcinoma, prostate adenocarcinoma, and endometrial carcinoma (97–99). Moreover, TBK1 expression is often silenced by promoter hypermethylation in cervical squamous carcinoma, kidney renal clear cell carcinoma, lung squamous carcinoma, and pancreatic adenocarcinoma, whereas the IRF3 promoter is more methylated in kidney renal clear cell carcinoma, lung squamous carcinoma, and pancreatic adenocarcinoma (98).

In an animal model, reversal of STING methylation in murine melanoma cell lines using a clinically available DNA methylation inhibitor improves agonist-induced STING activation and type I IFN induction, which induce tumor regression through a CD8^+^ T cell-dependent immune response (97). These findings not only provide mechanistic insight into how STING signaling dysfunction in tumor cells can contribute to impaired responses to STING agonist therapy but also suggest that pharmacologic restoration of STING signaling through epigenetic reprogramming enhances tumor immunogenicity, making tumors more vulnerable to T-cell cytotoxicity. Future studies should focus on developing reliable tools for patient stratification based on methylation status, confirming the functional restoration and safety of innate immune activation after therapy, and determining the most effective therapeutic combinations and regimens to harness epigenetic reprogramming in cancer treatment.

#### 4.1.2 Histone modifications

Beyond DNA methylation, histone post-translational modifications constitute another epigenetic layer that affects cGAS–STING signaling. The N-terminal tails of histone undergo diverse post-translational modifications (PTMs) such as methylation, acetylation, phosphorylation, and ubiquitination, which remodel chromatin architecture and regulate transcriptional accessibility at promoters and enhancers (100). Generally, histone acetylation catalyzed by histone acetyltransferases (HATs) promotes transcriptional activation, while deacetylation by histone deacetylases (HDACs) leads to repression. Likewise, histone methylation, which occurs primarily on lysine and arginine residues, is dynamically regulated by histone methyltransferases and demethylases, thereby exerting context-dependent effects on gene expression (101).

Emerging evidence links histone modifications to fine-tuning of the cGAS–STING pathway. For example, in microglia, histone deacetylase 3 (HDAC3) deacetylates NF-κB, p65, enhancing its nuclear translocation and binding to the cGAS promoter, thereby upregulating cGAS transcription (102). This epigenetic mechanism connects histone deacetylation with innate immune activation. Dysregulated HDAC3 activity could thus contribute to aberrant cGAS–STING signaling in certain diseases, including cancer, where chronic inflammation supports tumor progression. Conversely, histone deacetylation can also repress STING. In breast cancer cells, estradiol (via ERα) recruits HDAC3 to the STING promoter, inducing local deacetylation (e.g., loss of H3K4 acetylation) and silencing STING expression. Inhibition of HDAC3 reverses this repression, restoring STING levels and inhibiting tumor growth, and clustered regularly interspaced short palindromic repeats-associated protein 9 (CRISPR-Cas9) knockout of STING abolishes the antitumor effect of the HDAC3 inhibitor. This confirms that HDAC3 inhibitors suppress tumors at least in part by reactivating STING (103).

Histone methylation can likewise silence STING. In colon cancer cells, STING is frequently silenced through epigenetic repression mediated by lysine-specific demethylase 5 (KDM5) family enzymes. KDM5 demethylases remove methyl groups from histone H3 lysine 4 (H3K4), a mark associated with active transcription, thereby silencing STING expression. Inhibition of KDM5 with the compounds CPI-455 (*in vitro*) and CPI-48 (*in vivo*) restores STING transcription, thereby reactivating tumor cell-intrinsic antitumor immunity (104). In immunocompetent mice, the authors showed that CPI-48 treatment significantly suppressed the growth of wild-type MC38 colon cancer allografts but had no effect on STING-deficient tumors. This indicates that the antitumor activity of KDM5 inhibition is STING-dependent, and restoring STING expression via epigenetic blockade prevents immune evasion. Together, these findings illustrate how histone methylation, acetylation, and ubiquitination collectively regulate the cGAS–STING axis at both transcriptional and post-translational levels. This epigenetic control not only influences antiviral immunity but also shapes tumor progression and therapeutic responses, warranting further mechanistic studies to identify actionable targets.

### 4.2 Transcriptional regulation

Components of the cGAS–STING pathway are tightly regulated at the transcriptional level. Mapping of the human cGAS promoter revealed that the minimal core promoter lies within −414 to +76 nucleotides (nt) relative to the transcription start site. Functional assays demonstrated that transcription factors Specificity Protein 1 (Sp1) and cAMP response element-binding protein (CREB) bind directly to this region and are essential for basal promoter activity, as their mutations or knockdowns markedly suppressed cGAS expression, whereas their overexpression enhanced it (105). These results indicate that cGAS activity is regulated not by frequent genetic alterations but rather by transcriptional control by specific DNA-binding proteins, which are themselves modulated by post-translational modifications and signaling cues. Mechanistically, this positions cGAS expression as highly responsive to upstream cellular states, with important implications for innate immune signaling, antiviral defense, and cancer biology, where dynamic regulation rather than gene mutation likely drives cGAS pathway activity.

### 4.3 Post-translational modification

Beyond genetic and transcriptional control, cGAS activity is tightly regulated by PTMs. A variety of covalent modifications of cGAS or STING can enhance or inhibit signaling by altering protein enzymatic activity, stability, localization, or interactions. These modifications allow the cell to fine-tune the pathway’s activity in response to different stimuli and to maintain homeostasis. Major post-translational regulators of cGAS–STING include palmitoylation, ubiquitination, acylation, phosphorylation, and other modifications, as summarized subsequently.

#### 4.3.1 Palmitoylation

Palmitoylation is a reversible lipid modification in which fatty acids, most commonly palmitic acid, are covalently attached to cysteine residues of proteins. This modification, catalyzed by a family of DHHC-type zinc-finger-containing (ZDHHC) palmitoyltransferases, can alter a protein’s stability, localization, and interactions, thereby exerting broad influence on cellular signaling (106). Many cytoplasmic and membrane-associated proteins rely on palmitoylation for proper function. During STING signaling, STING itself is palmitoylated at cysteine residues Cys88 and Cys91 at the trans-Golgi network (TGN), a modification essential for its multimerization within lipid rafts and for the efficient recruitment of TBK1 and IRF3, thereby promoting a robust type I interferon response (107, 108). Indeed, inhibition of STING palmitoylation (or mutation of the palmitoylation cysteines) disrupts STING’s signaling capacity, underscoring that this lipid modification is critical for full innate immune activation.

#### 4.3.2 Ubiquitination

Ubiquitination is a versatile PTM whereby ubiquitin chains are attached to lysine residues on a target protein, influencing its activity or stability. STING undergoes complex ubiquitination that can either promote its activation (by aiding IRF3 recruitment and Golgi translocation) or mark it for proteasomal degradation, depending on the linkage type and residue (108–111). cGAS is also ubiquitinated. The monoubiquitination of cGAS by E3 ligases such as tripartite motif-containing protein 56 (TRIM56) and TRIM41 enhances cGAS dimerization, DNA binding, and cGAMP production, thereby promoting innate immune activation (112). In contrast, polyubiquitination, especially K48-linked chains, marks cGAS and STING for proteasomal degradation, ultimately dampening the signaling and facilitating immune resolution (113). K27-linked or K63-linked polyubiquitination, catalyzed by E3 ligases like RNF185 and TRIM56, activates cGAS and STING by supporting their oligomerization, trafficking, and the recruitment of downstream effectors like TBK1 and IRF3 for interferon production. Deubiquitinases (e.g., USP14, USP27X, and USP21) counteract degradation by removing K48-linked chains, stabilizing both cGAS and STING, and sustaining antiviral responses (114). The dysregulation of this ubiquitin machinery can lead to excessive or insufficient interferon signaling and is implicated in diverse conditions, including autoimmunity, cancer, and viral infections, where targeting these modifications could refine immunotherapeutic approaches.

#### 4.3.3 Acetylation

The activation of cGAS is tightly controlled by acetylation at multiple lysine residues, with distinct acetylation events exerting either inhibitory or activating effects on cGAS function. In resting states, human lysine residues are acetylated at Lys384, Lys394, and Lys414 to suppress their activation and prevent inappropriate immune responses (115). Upon DNA challenge, deacetylation at these sites relieves this inhibition, enabling cGAS activation. Conversely, acetylation by the lysine acetyltransferase KAT5 at N-terminal lysines (Lys47, Lys56, Lys62, and Lys83) enhances cGAS’s ability to bind DNA, promoting activation (116). Notably, aspirin can directly acetylate cGAS at K384 and/or K394 and K414, thereby inhibiting its activation, highlighting its potential therapeutic application for autoimmune diseases (44). Evidence from preclinical models suggests that aspirin is a potential agent in disease management. In Three Prime Repair Exonuclease 1 −/− (Trex1−/−) mice, a model of Aicardi–Goutières syndrome (AGS), aspirin increases cGAS acetylation, reduces ISG expression, and improves survival (115). Similarly, aspirin treatment of peripheral blood mononuclear cells (PBMCs) from an AGS patient with a TREX1 mutation significantly lowered ISG levels, demonstrating its therapeutic potential in human autoimmune disease. These findings suggest that aspirin could be repurposed to target cGAS-driven pathologies, including self-DNA-induced autoimmunity and type I interferonopathies, by modulating post-translational acetylation and immune signaling (115). Further proteomics studies have revealed additional acetylation sites such as Lys198 and Lys414, with acetylation at Lys198 promoting activation and Lys414 suppressing it, and viral infections like herpes simplex virus type 1 (HSV-1) and human cytomegalovirus (HCMV) manipulate these modifications to evade immune detection (117).

Taken together, acetylation integrates into a broader network of cGAS post-translational modifications, including phosphorylation, ubiquitination, and SUMOylation. These modifications collectively fine-tune cGAS activity across different physiological states, maintaining repression in homeostasis, enabling rapid activation upon DNA sensing, and preventing inappropriate activation during cell cycle phases exposed to naked DNA. Understanding the spatial and temporal coordination of these modifications remains a major frontier, with significant implications for both antiviral defense and immune-targeted therapies.

#### 4.3.4 Phosphorylation

Protein phosphorylation, dynamically regulated by kinases and phosphatases, fine-tunes cGAS–STING signaling, balancing antiviral defense with the risk of autoimmunity. cGAS phosphorylation provides a checkpoint for DNA sensing, with multiple kinases suppressing cGAS activity by phosphorylating distinct residues. For instance, phosphorylation of cGAS by AKT and CDK1 at S291/S305 during mitosis, DNA-dependent protein kinase (DNA-PK) at T68/S213, and B lymphoid tyrosine kinase (BLK) at Y215 suppresses the enzymatic activity of cGAS (118–120). Since the PI3K/AKT pathway is commonly hyperactivated in human cancers, where it contributes to immune evasion, it is plausible that tumors exploit AKT-mediated cGAS inhibition to suppress cGAS activity and establish an immune microenvironment that supports tumor progression (121).

STING is phosphorylated by the kinase TBK1, a process that is essential for initiating downstream immune signaling. TBK1 specifically targets the C-terminal tail of STING at Ser366, allowing STING to recruit the transcription factor IRF3 and enable its phosphorylation (18). Once phosphorylated, IRF3 dimerizes and translocates into the nucleus, where it drives the transcription of type I interferon genes (such as IFN-β) and mounts potent antiviral and antitumor responses. Conversely, phosphatases provide an essential counterweight. Protein phosphatase, Mg^2+^/Mn^2+^-dependent 1A can antagonize STING aggregation by dephosphorylation of both STING and TBK1, thereby inhibiting STING-induced antiviral signaling (122). Together, kinases and phosphatases thus serve as crucial molecular switches, and their dysregulation may link innate immune signaling to viral persistence, cancer progression, and autoimmune disease.

#### 4.3.5 Glutamylation

Glutamylation represents a recently identified layer of post-translational regulation of cGAS, adding complexity to the control of innate immune signaling. A seminal discovery by Xia and colleagues shows that cGAS is regulated by glutamylation, catalyzed by tubulin tyrosine ligase-like glutamylases (TTLL4 and TTLL6), and reversed by cytosolic carboxypeptidases (CCP) such as CCP5 and CCP6 (123). TTLL6 mediates polyglutamylation at Glu272, which inhibits cGAS’s ability to bind DNA, while TTLL4 catalyzes monoglutamylation at Glu302, blocking its synthase enzymatic activity for cGAMP synthesis. CCP5 and CCP6 deglutamylate these sites, thereby restoring DNA binding and enzymatic function (123). Further studies have shown that mice deficient in CCP5 or CCP6 show impaired type I interferon production and increased susceptibility to DNA virus infections, highlighting the critical role of these modifications in antiviral immunity. Although the precise regulatory mechanisms that trigger these enzymes remain unknown, these findings suggest that a dynamic glutamylation fine-tunes cGAS activity and may be relevant in cancer, where altered immune surveillance and DNA sensing are critical for tumor progression and immune evasion.

#### 4.3.6 SUMOylation

The addition of small ubiquitin-like modifier proteins is another post-translational regulatory mechanism (PRM) that impacts cGAS–STING. SUMOylation dynamically regulates cGAS–STING signaling, impacting the stability and function of both cGAS and STING during antiviral responses. It was found that TRIM38-mediated SUMOylation of cGAS at K217 in resting cells stabilizes the protein by blocking K48-linked polyubiquitination, whereas infection-induced SUMOylation at K464 prevents premature degradation during early viral sensing (124). Interestingly, this SUMOylation is dynamic: during later phases of infection, the SUMO protease SENP2 deconjugates SUMO from cGAS and STING, leading to their degradation via proteasomal and autophagic pathways, thereby preventing excessive immune activation (124). This observation suggests that dysfunction in this SUMOylation–deSUMOylation balance may lead to impaired antiviral immunity and exaggerated inflammatory conditions.

### 4.4 Regulation by metal ions

The activity of cGAS is strongly influenced by several ions, such as Zn^2+^, Mn^2+^, Co^2+^, Co^2+^ and K^+^ ions (125, 126). For example, Zn^2+^ promotes cGAS phase transition with DNA (125), while K^+^ efflux appears to suppress cGAS activation (126), and dysregulated ion transport in cancer may modulate cGAS signaling to favor immune evasion. Notably, Mn^2+^ regulates anti-cancer immunity primarily by activating the cGAS–STING pathway, which is essential for innate immune sensing of tumors. By promoting DC and macrophage maturation, Mn^2+^ enhances tumor-specific antigen presentation, leading to stronger CD8^+^ T-cell differentiation, activation, and memory formation (127). Remarkably, further *in vivo* melanoma models showed that Mn^2+^-insufficient mice showed increased tumor growth and metastasis with reduced tumor-infiltrating CD8^+^ T cells, highlighting its critical role in antitumor immunity. Importantly, combining Mn^2+^ with immune checkpoint inhibitors synergistically improves therapeutic efficacy, reduces required anti-programmed cell death protein 1 (anti-PD-1) doses, and shows promising results in clinical trials by inducing type I IFNs and reviving immunotherapy responses. Despite these promising findings, key knowledge gaps remain, including how Mn^2+^ selectively enhances tumor versus normal tissue antigen presentation, the optimal dosing and delivery strategies for maximal therapeutic effect, and its long-term efficacy and safety across diverse cancer types. Addressing these gaps will be critical for translating Mn^2^-based strategies into widely effective cancer immunotherapies.

### 4.5 Integrated regulation of cGAS–STING signaling governs immune homeostasis and disease

Emerging evidence indicates that post-translational modifications of cGAS and STING do not act in isolation but instead form an integrated regulatory network that collectively determines signaling threshold, duration, and output specificity. A clear example of this coordination is provided by TBK1-dependent phosphorylation of the autophagy adaptor p62 following cGAS–STING activation, which enables p62 to selectively recognize ubiquitinated STING and target it for autophagosomal degradation. This cooperative interaction between phosphorylation and ubiquitination establishes an intrinsic negative feedback loop that restrains prolonged STING signaling once activation has occurred (128). In parallel, additional layers of crosstalk have been described at the level of cGAS itself, where acetylation modulates its enzymatic activity and DNA-binding capacity and appears to be functionally linked to its ubiquitination status (129–131). These interconnected modifications fine-tune cGAS activity and downstream type I interferon production, highlighting how PTM crosstalk governs both the amplitude and persistence of innate immune signaling.

The disruption of this coordinated PTM landscape can therefore shift cGAS–STING signaling from tightly controlled host defense toward pathological immune activation or inappropriate immune suppression. The aberrant hyperactivation of the cGAS–STING pathway has been implicated in the pathogenesis of multiple autoimmune and autoinflammatory diseases, including AGS, rheumatoid arthritis, and systemic lupus erythematosus (SLE). In these conditions, sustained type I interferon and pro-inflammatory cytokine production drives chronic immune activation and tissue damage, whereas their inhibition increases susceptibility to infections (132, 133).

SLE provides a clear example of how dysregulated PTM crosstalk at the cGAS level lowers the activation threshold of cytosolic DNA sensing. In SLE, the E3 ubiquitin ligase RNF185 is upregulated and promotes K27-linked ubiquitination of cGAS, enhancing its enzymatic activity and cGAMP production. This amplifies STING–TBK1–IRF3 signaling and sustains type I interferon and ISG expression (113). By acting upstream of STING rather than as an interferon-inducible feedback regulator, RNF185 contributes to the chronic interferon signature and systemic inflammation in SLE. Conversely, opposing PTMs can impose inhibitory constraints on cGAS activity, illustrating how an imbalance between activating and repressive modifications determines disease outcome. cGAS is a direct substrate of the serine/threonine kinase AKT, which phosphorylates cGAS at S291 in mice (S305 in humans), thereby suppressing its enzymatic activity and attenuating downstream innate immune signaling. Pharmacologic inhibition of AKT using AKT inhibitor VIII, as well as expression of a non-phosphorylatable cGAS S291A mutant, enhanced DNA-induced IFN-β production and restricted HSV-1 infection, highlighting AKT-mediated phosphorylation as a negative regulatory checkpoint of cGAS activity (119).

Moreover, dysregulated metal ions can alter cGAS–STING activity and contribute to disease. For example, Mn^2+^ deficiency reduces cGAS–STING signaling, impairing dendritic cell maturation and CD8^+^ T-cell responses, which promotes tumor growth and metastasis in mouse models (127, 134). Conversely, excessive or mislocalized Mn^2+^ and Zn^2+^ can aberrantly activate cGAS–STING and inflammasome pathways, driving chronic inflammation and potentially contributing to autoimmune or neuroinflammatory diseases (135). Thus, metal ion imbalance can both suppress antitumor immunity and promote pathological inflammation via cGAS–STING dysregulation.

## 5 Experimental and preclinical evidence for the therapeutic roles of cGAS–STING activation

Consistent with clinical observations the activation of the STING pathway in several preclinical models has been shown to induce strong antitumor immunity, engaging both the innate and adaptive arms of the immune system, reducing tumor burden, and establishing long-term immunologic memory. These experimental systems provide powerful platforms to dissect the mechanistic roles of STING signaling in tumor initiation, progression, and therapeutic response. In the following section, we highlight several preclinical cancer models in which STING activation induces significant antitumor effects. This evidence provides a mechanistic basis upon which clinical trials can be developed to leverage STING pathway activation as part of next-generation immunotherapeutic regimens.

### 5.1 Melanoma

Melanoma is one of the cancers in which STING is frequently inactivated through promoter hypermethylation (98). Falahat et al. (97) showed that the intratumoral administration of the STING agonist delays tumor growth only in wild-type mice. Still, they had no impact on tumors lacking STING, demonstrating that melanoma-intrinsic STING is essential for early-phase antitumor responses and robust CD8^+^ T-cell infiltration (97). However, this effect was not durable, as STING agonist monotherapy failed to maintain long-term tumor control, indicative of adaptive resistance to STING signaling. Interestingly, combining DNA methyltransferase inhibitor and STING agonist results in synergistic tumor regression, increased tumor T-cell homing signal CXCL10, and augmented infiltration of CXCR3^+^ CD8^+^ T cells (97). Importantly, this therapeutic synergy is abrogated by CD8^+^ T-cell depletion, confirming the T cell-dependent nature of the antitumor response.

In a recent study, the intratumoral delivery of the STING agonist 5,6-dimethylxanthenone-4-acetic acid (DMXAA) into a syngeneic mouse model resulted in the complete regression of B16.SIY melanoma tumors in wild-type mice, but not in STING-deficient counterparts (4). Mechanistically, this tumor clearance was associated with the increased recruitment of antigen-presenting cells and the expansion of tumor-specific IFN-γ-producing CD8^+^ T cells, responses that were absent in STING^−/−^ and IFNAR^−/−^ mice, thereby indicating a type I interferon-dependent adaptive immune response (4). These antitumor effects were lost in rag2-deficient and CD8^+^-depleted mice, underscoring the indispensable role of CD8^+^ T cells in mediating tumor regression. Remarkably, DMXAA treatment also induced durable immunologic memory, as rechallenged mice remained resistant to tumor growth for up to 60 days after initial clearance. Even more compelling, localized STING activation triggered regression of distant, untreated tumors, demonstrating a systemic therapeutic effect mediated by activated T cells rather than drug diffusion. This abscopal effect was absent following systemic DMXAA administration, highlighting the need for localized STING activation within the TME. Further experiments confirmed robust tumor suppression across multiple murine tumor models (B16.F10, TRAMP-C2, 4T1, and Ag104L), suggesting that the efficacy of STING agonists is not restricted to a single tumor type (4). Collectively, these findings demonstrate that acute, localized STING activation can induce curative, CD8^+^ T cell-driven immunity and provide a strong translational rationale for developing STING agonists as cancer immunotherapies in melanoma.

### 5.2 Ovarian cancer

Ovarian cancer (OC) is a heterogeneous group of malignancies arising from distinct cell types within the ovary. The most common and lethal subtype is high-grade serous ovarian cancer (HGSOC), accounting for approximately 70% of cases, characterized by frequent DNA repair defects such as BRCA1/2 mutations (136). DNA damage and chromatin instability are hallmarks of many ovarian cancers (137), positioning the cGAS–STING pathway as a crucial mediator of tumor immunogenicity and therapy response.

In BRCA1-deficient HGSOCs, the resultant chromatin instability leads to the cytosolic accumulation of double-stranded DNA (dsDNA), which activates STING (138). This activation induces type I IFN signaling, promotes CD8^+^ T-cell infiltration, and enhances tumor immunogenicity to ovarian tumors. However, this observation is nuanced by the observation that intrinsic escape mechanisms counterbalance BRCA1mut tumors that are “locked” in a pro-inflammatory STING signature. These include the downregulation of CCL5 (via chromosomal alteration or methylation) and the loss of DNA-sensing/IFN genes such as IFNB1 and NF-κB1, which blunt STING-driven inflammation and allow immune escape (138). Moreover, the long-term activation of STING promotes VEGF-A expression, directly linking angiogenesis as another layer of resistance mechanism from chronic STING activation. Interestingly, genetic ablation and pharmacologic VEGF-A blockade in these models enhance T-cell infiltration, suppress neovascularization, and restore sensitivity to immune checkpoint blockade (ICB) or combination ICB/PARP inhibitor therapy. These effects depend solely on tumor-intrinsic STING activity and the homologous recombination deficiency (HRD) genotype. This study provides insights into the pleiotropic roles of STING in promoting tumor-intrinsic mechanisms of both immunoreactivity and immune resistance.

Furthermore, as reported, clinical cisplatin treatment in ovarian cancer models activates the DNA‐sensing cGAS–STING pathway by inducing cytosolic DNA damage, which upregulates both cGAS and STING protein levels and, in turn, drives increased PD-L1 and MHC-I expression on tumor cells. The direct stimulation of STING with cGAMP or interferon-stimulatory DNA recapitulates these effects, confirming that STING signaling boosts tumor immunogenicity while also triggering adaptive resistance via PD-L1. *In vivo*, cisplatin combined with PD-L1 blockade prolongs survival in cisplatin-sensitive and moderately resistant tumors and remodels the TME toward a more cytotoxic profile, although the survival benefit is modest in highly resistant models. A key strength of these studies is the integration of RNA-seq, protein analysis, and functional assays to trace a clear mechanistic axis from DNA damage to immune modulation. However, limitations include reliance on overexpression systems and a lack of STING knockout or pharmacologic inhibition studies *in vivo* to prove causality definitively. It remains unknown whether these cisplatin–STING interactions occur in patient tumors and how best to exploit them clinically, particularly which combinations or sequencing with immunotherapies will yield durable responses (139).

### 5.3 Endometrial cancer

The cGAS–STING pathway plays a multifaceted role in modulating tumor behavior and immune responses in endometrial cancer yet remains underexplored compared to its investigation in other gynecological malignancies (140). Emerging evidence implicates the dysregulation of the cGAS–STING pathway in tumor progression and immune evasion. For instance, S100A2, a marker of aggressive endometrial tumors, has been shown to promote tumor aggressiveness through various mechanisms, including the suppression of the GAS–STING pathway (140). Similarly, epigenetic silencing of STING has been reported in endometrial cancer (EC). A recent paper found that HDAC3 suppresses STING expression epigenetically by deacetylating histone H3 at the STING promoter, an effect driven by ERα signaling (103). This study further demonstrated that inhibition of HDAC3 reactivates STING, leading to increased apoptosis and tumor suppression. Together, these findings suggest that a well-regulated cGAS–STING axis can suppress tumor progression, but its dysregulation may contribute to immune escape. Hence, targeting epigenetic regulators or oncogenic repressors of STING represents a promising therapeutic strategy for re-engaging innate immunity in endometrial cancer.

### 5.4 Colon cancer

Consistent with human colorectal tumors, where more than 20% of late-stage colon cancers exhibit low expression of STING (36), CRISPR/Cas9-mediated knockout of STING in mouse colon cancer cell lines (MC38 and CT26) significantly accelerated tumor growth in immunocompetent mice, indicating a role for STING in modulating host immunity (104). Interestingly, depletion of CD4^+^, CD8^+^ T cells, and NK cells abolished the growth difference between STING knockout and control tumors, suggesting that STING in tumor cells acts by activating cytotoxic lymphocytes. While the total number of infiltrating immune cells was unchanged, STING-deficient tumors exhibited reduced expression of functional activation markers (granzyme B, CD69, and IFN-γ) on both CD8^+^ T cells and NK cells, indicating impaired effector function. Moreover, since STING is frequently silenced in colorectal cancer, likely via epigenetic repression by KDM5 demethylases (141), the possibility of pharmacologic inhibition of KDM5 is being explored. This approach restored STING expression and suppressed tumor growth *in vivo*, but only in tumors with functional STING. These results indicate that tumor-intrinsic STING signaling promotes antitumor immunity by enhancing the activation of CD8^+^ T cells and NK cells and that pharmacologic restoration of STING may serve as a promising immune-modulating strategy for colorectal cancer therapy.

Another study by Ahn et al. (2015) showed that STING-deficient mice displayed increased susceptibility in the azoxymethane (AOM)/dextran sulfate sodium (DSS)-induced colon cancer mouse model (142). Mechanistically, in colitis, the STING pathway-dependent activation of IRF3 and NF-κB triggers innate immunity-related inflammatory responses and immune cell infiltration. However, in colon cancer, the activation of the STING pathway alleviates the immunosuppressive TME and overcomes resistance to tumor immunotherapy, resulting from inadequate cytotoxic T-cell responses. Notably, combining the STING agonist with anti-PD-1 therapy significantly enhanced therapeutic efficacy, resulting in the dramatic tumor regression, robust activation of CD8^+^ T cells, and increased inflammatory macrophage infiltration (143). These results demonstrate that STING and PD-1 blockade synergistically remodel the TME and promote effective antitumor immunity in peritoneal colon cancer. Furthermore, Liang and colleagues recently provided indirect yet compelling evidence for the roles of STING activation for durable tumor control in prostate cancer. This group showed that riluzole induced tumor suppression in syngeneic immunocompetent mice in a STING-dependent manner. This activation resulted in enhanced CD8^+^ T-cell infiltration and improved response to anti-PD-1 therapy (144). The loss of STING abolishes these effects, underscoring its essential role in linking DNA damage to immune activation and sensitizing tumors to immune checkpoint blockade.

### 5.5 Prostate cancer

Recent evidence from the prostate cancer model demonstrates that tumor cell-intrinsic STING expression is essential for mediating the antitumor effects of STING agonists. In this model, STING-deficient TRAMP-C2 cells exhibited impaired IFN-I responses and grew significantly faster in immunocompetent mice compared to their STING-sufficient counterparts (145). The intratumoral administration of the STING agonist cGAMP suppressed tumor growth only in STING-expressing tumors, confirming that tumor-intrinsic STING signaling is necessary for the anti-cancer activity of cGAMP in this context. Mechanistically, STING activation enhanced immune cell infiltration into the TME, and STING-sufficient tumors exhibited higher infiltration of CD45^+^ leukocytes and CD11c^+^ dendritic cells, both of which were further increased by cGAMP treatment in a STING-dependent manner. Importantly, CD8^+^ cytotoxic T cells were also significantly enriched in STING-expressing tumors following cGAMP administration, whereas STING-deficient tumors failed to recruit these cells. These findings suggest that tumor-intrinsic STING facilitates immune-mediated tumor control by recruiting and activating antigen-presenting cells and cytotoxic lymphocytes (145).

### 5.6 Glioblastoma

Despite the transformative impact of immunotherapy in several solid malignancies, glioblastoma (GBM) remains notoriously refractory to immune-based treatments (146). This is largely due to its profound immunosuppressive microenvironment, whereby spontaneous antitumor T-cell responses are either absent or suppressed (147, 148). One emerging strategy to invigorate antitumor immunity in GBM is the activation of the STING pathway, given its role as a key upstream mediator of IFN-I signaling. However, STING signaling is epigenetically silenced in GBM tumor cells primarily via promoter methylation, although its expression remains intact in glioma-associated myeloid and vascular stromal cells, which may respond to STING agonists. Importantly, preclinical models have demonstrated that STING agonism in intracranial GBM triggers robust innate immune activation, characterized by macrophage, neutrophil, and NK cell infiltration, although the precise contribution of each immune subset to tumor control remains to be fully delineated.

Although STING agonists for patients with infiltrating gliomas have not yet entered human clinical trials, initial promising results have been reported in animal models. For instance, injection of the STING agonist c-di-GMP into the tumors of glioma-bearing mice significantly improved survival, enhanced IFN-I signaling, upregulated T-cell homing chemokines (Ccl5 and Cxcl10), and increased T-cell migration into the brain (149). These effects were not observed in mice homozygous for the non-functional STING variant, establishing the necessity of STING expression in the TME. Additionally, the combination of c-di-GMP and an ovalbumin-targeted peripheral vaccine significantly increased survival in glioma-bearing mice compared with monotherapy with either c-di-GMP or the peripheral vaccine alone.

Recent evidence also indicates that STING signaling is required for durable antitumor effects related to HSV-1 (150). However, as discussed above, primary brain tumors lack STING expression and exhibit hypermethylation of a region of the STING promoter (151). Interestingly, STING expression and signaling can be reconstituted in glioma cell lines by exposure to decitabine, a DNA hypomethylating agent that enhances immune recognition and killing of glioma-initiating cells (152). Given the established role of STING signaling in cancer immunity and the potential to modulate or reconstitute it in neoplastic cells, rationally targeting this axis may yield therapeutic benefits when combined with oncolytic herpes simplex viruses and/or radiotherapy. As such, therapeutic strategies aiming to restore tumor-intrinsic STING signaling include demethylating agents such as decitabine, which reverse promoter methylation and restore STING expression in GBM cell lines, as well as the direct administration of synthetic STING agonists. Another parallel study investigated the role of STING in GBM, demonstrating that STING is expressed in human GBM tissue, particularly within the tumor vasculature, and that the stimulation of GBM explants with the STING agonist triggered robust inflammatory cytokine release. In murine GBM models (GL261 and CT-2A), the intracranial administration of ADU-S100 via biodegradable implants profoundly remodeled the TME, leading to substantial infiltration of innate immune cells, including macrophages, neutrophils, and NK cells, and significantly prolonged survival (153). Notably, the antitumor effects were abrogated by NK cell depletion, indicating a key role for STING-induced NK cell-mediated tumor clearance. These findings underscore the therapeutic potential of STING activation to overcome immunologic resistance in GBM and provide a strong rationale for combining STING agonists with other modalities, such as checkpoint blockade, cancer vaccines, or NK-based therapies.

### 5.7 Pancreatic ductal adenocarcinoma

PDAC represents the most common pancreatic cancer (154) and bears the poorest known clinical outcome among solid tumors, with a 5-year survival rate <10%. This dismal prognosis stems largely from the limited efficacy of current standard-of-care, including chemotherapies and immunotherapies (155). Notably, the transformative success of immunotherapies across various malignancies has not been replicated in PDAC, primarily attributed to the profoundly immunosuppressive TME (156), which poses a substantial barrier to adequate immune-mediated tumor clearance. As a result, overcoming this suppressive TME remains an attractive strategy for improving immunotherapy responses in PDAC (156).

Several preclinical studies have demonstrated that STING agonists, such as DMXAA, induce robust antitumor responses in both orthotopic and subcutaneous murine models of pancreatic cancer, particularly when combined with chemotherapeutics like gemcitabine. Mechanistically, STING activation reprograms tumor-associated macrophages toward a pro-inflammatory phenotype, promotes DC activation, and enhances CD8^+^ T-cell infiltration, ultimately leading to robust tumor regression (156–158). These findings suggest a beneficial role for the activation of the cGAS–STING pathway in restraining pancreatic cancer progression. However, there is an important nuance to this narrative: recent research has shown that STING activation may induce regulatory IL-35^+^ B cells that suppress NK cell activity and impair NK cell-mediated cytotoxicity (76). Given that pharmacologic and genetic blockade of IL-35 in B cells reduces tumor growth, a combinatorial strategy to overcome immunosuppression in tumors may be necessary.

Beyond the DC–CD8^+^ T-cell axis, research has indicated that the stimulation of pancreatic cancer cells with the STING agonist cGAMP upregulates NKG2D ligands and chemokines such as CCL5 and CXCL10, which recruit and activate NK cells in the TME. This enhances the sensitivity of pancreatic cancer cells to NK cell-mediated cytotoxicity (159). Combining cGAMP with mesothelin-targeted CAR-NK cells synergistically improves antitumor effects both *in vitro* and *in vivo*. These findings suggest that cGAMP may serve as a novel adjuvant to potentiate NK cell therapies in pancreatic cancer treatment.

Cancer-associated fibroblasts (CAFs) in PDAC contribute to poor immune infiltration by creating a dense fibrotic stroma that physically blocks immune cells. They also secrete immunosuppressive chemokines, cytokines, and growth factors that recruit regulatory T cells, MDSCs, and TAMs, which suppress cytotoxic CD8^+^ T-cell activity (160–162). This leads to an immunosuppressive TME and poor responses to immunotherapy. Despite these insights, targeting CAFs therapeutically has been challenging due to their heterogeneity and lack of unifying markers. Interestingly, emerging reports have indicated that activating the cGAS–STING pathway can overcome the immunosuppressive environment orchestrated by CAFs. A 2022 study by a Japanese group showed that the activation of cGAS–STING signaling in cancer cells supports the maintenance of tumor-suppressive features in CAFs, as evidenced by the reduced expression of markers such as Meflin and TIMP-1 when cGAS is absent (163). Extending these findings to human PDAC specimens, cGAS-negative patients had significantly worse survival than cGAS-positive patients. Importantly, patients double-positive for both cGAS and STING showed improved survival compared to non-D-positive patients. Multivariate analysis further confirmed that the co-expression of cGAS and STING independently correlated with better survival outcomes after adjusting for age and stage.

Moreover, a recent study employing single-cell RNA sequencing in PDAC has identified a novel CAF subset termed interferon-response CAFs (ifCAFs), characterized by the expression of inflammatory genes (*Rsad2*, *Ifit3*, *Cxcl10*, and *Ddx58*) and enrichment for type I interferon signaling (164). STING agonism reprograms CAFs from a tumor-promoting myCAF phenotype to ifCAFs that promote neutrophil polarization toward tumor suppression. In orthotopic tumor models, oral STING agonist increases ifCAF abundance and IFN-I signaling, fostering an antitumor microenvironment by enhancing tumor-suppressive neutrophils and CD8^+^ T-cell cytotoxicity. This suggests stromal reprogramming as a next-generation strategy, in contrast to broad CAF ablation, which has yielded paradoxical outcomes. However, successful clinical translation must address tumor heterogeneity, dynamic CAF plasticity, the identification of predictive biomarkers for patient selection, and the long-term consequences of altering stromal–immune interactions. Identifying biomarkers and understanding long-term impacts are essential to harnessing STING agonists for durable cancer control.

### 5.8 Hepatocellular carcinoma

The activation of the STING pathway plays a crucial role in suppressing HCC progression through several mechanisms, including promoting tumor cell apoptosis, autophagy, and IFN-I responses. In a DEN-induced mouse model of HCC, STING deficiency facilitates an aggressive tumor, highlighting a potential role of cGAS–STING in restraining tumor growth (165). Consistently, systemic treatment with the STING agonist 3′3′-cAIMP reduced tumor size, increased intratumoral cleaved caspase-3, and promoted CD8^+^ T-cell infiltration, indicating enhanced antitumor immunity. Mechanistically, STING activation in Kupffer cells promotes the presentation of tumor antigens and the secretion of cytokines (e.g., TNF-α and IL-12), which recruit and activate cytotoxic CD8^+^ T cells to the TME, supporting adaptive immune responses. Genetic ablation of STING impairs immune activation signals (like STAT1/3 phosphorylation) and autophagy, further contributing to tumor progression. Thus, STING pathway activation modulates macrophage-mediated innate and CD8^+^ T-cell adaptive immunity, creating a tumor-suppressive microenvironment that can limit HCC tumorigenesis and offers a promising therapeutic target, especially when combined with other immunotherapies.

In another study, a genotoxic agent, CFI-402257, was shown to induce the accumulation of cytosolic DNA in HCC. This aberrant DNA is sensed by cGAS, leading to the activation of the STING signaling pathway. Transcriptomic analyses revealed that CFI-402257 induces a broad SASP secretome, such as CCL2 and IL-1β, in a STING-dependent manner (166). The secreted SASP factors remodel the TME by enhancing the infiltration and activation of effector CD4^+^ and CD8^+^ T cells, as well as NK cells, while concomitantly reducing immunosuppressive regulatory T cells. Notably, these immunostimulatory effects and antitumor responses are abolished in STING-deficient HCC models, underscoring the essential role of intact cGAS–STING signaling in SASP-mediated immune remodeling and tumor suppression. To explore therapeutic synergy, a Trp53 knockout and Myc overexpression (Trp53KO/MycOE) murine HCC model generated by hydrodynamic tail vein injection was treated with CFI-402257 alone, anti-PD-1 immune checkpoint blockade alone, or their combination. The combined regimen markedly prolonged survival compared to either monotherapy, suggesting that mitotic checkpoint inhibition can potentiate immune checkpoint blockade efficacy by engaging cGAS–STING-mediated immunomodulation in the TME.

In a separate study, the mitotic checkpoint kinase inhibitor CFI-402257 was shown to induce cytosolic DNA accumulation specifically in HCC cells. This aberrant DNA is sensed by cGAS, which activates the STING signaling pathway and triggers a robust SASP. Transcriptomic analyses revealed a broad induction of SASP factors, including CCL2 and IL-1β, in a STING-dependent manner (166). These secreted cytokines reshape the TME by promoting the infiltration and activation of effector immune cells such as CD8^+^ T cells and NK cells while concurrently diminishing the presence of immunosuppressive Tregs. Crucially, the immunostimulatory milieu and resultant antitumor immunity elicited by CFI-402257 were abolished in STING-deficient HCC models, underscoring the essential role of intact cGAS–STING signaling for SASP-mediated immune remodeling and tumor suppression. *In vivo* experimental evidence showed that the antitumor efficacy of CFI-402257 in HCC is, at least in part, dependent on STING signaling. While CFI-402257 markedly prolonged survival in STING wild-type mice, this benefit was nearly abolished in STING-deficient mice, indicating that STING is critical for mediating its therapeutic effect. Moreover, combining CFI-402257 with anti-PD-1 immune checkpoint blockade significantly extended survival beyond either monotherapy, suggesting a synergistic interaction between STING activation and T cell-based immunotherapy. Collectively, these findings demonstrate that the STING pathway serves as a molecular bridge between genotoxic stress and antitumor immune responses in HCC, with the activation of the SASP playing a crucial role in orchestrating robust tumor immune surveillance and suppression.

## 6 Prognostic, immunologic, and therapeutic implications of cGAS–STING pathway across human cancers

Recent research has revealed that many human tumors retain functional cGAS–STING signaling, although the degree of activation varies substantially across cancer types. In most malignancies, elevated tumor mutational burden (TMB) and genomic instability serve as primary drivers of cGAS–STING activation by generating cytosolic DNA that triggers innate immune sensing. However, additional mechanisms, including epigenetic silencing, viral immune evasion, and oncogene-driven modulation, can either suppress or aberrantly sustain pathway activity. Consequently, the clinical impact of cGAS–STING signaling is highly context-dependent. In some cancers, elevated STING pathway expression is associated with enhanced tumor immunogenicity, increased immune infiltration, and favorable prognosis or improved responsiveness to immunotherapy. In contrast, chronic or dysregulated activation may promote tumor progression through inflammation, immune exhaustion, or stromal remodeling. To capture these multifaceted roles, **Table 1** synthesizes current evidence from literature and pan-cancer datasets, summarizing expression patterns, prognostic significance, immune-TME features, immunotherapy responsiveness, and key mechanistic determinants across major cancer types.

**Table 1:** Prognostic, immunologic, and therapeutic implications of cGAS–STING pathway across human cancers.

Cancer type	Detection method/biomarkers	STING status/expression	Prognostic impact	TME characteristics	IMT/treatment response	Mechanistic notes	References
Skin cutaneous melanoma (SKCM)	IRF3 level using Western blotting. CXCL10 and IFN-β detected with ELISA	↓ (often silenced by promoter hypermethylation)	↑ cGAS/STING often → improved survival (context-dependent)	↑ TILs, altered stroma; immune-inflamed in some subtypes	Better response to ICIs (e.g., ipilimumab) and BRAF inhibitors in STING-high tumors	Promoter methylation can silence STING activity; demethylating agents may restore STING and MHCI/II expression	(167–169)
Uveal Melanoma	cGAS expression level measured using *in silico* (TCGA)	↑ cGAS (distinct pattern)	Associated with poorer survival	Altered stromal composition; immune infiltration patterns differ from SKCM	Limited benefit from ICIs historically	High cGAS linked to immune suppression in uveal subtype	(168)
Non-small cell lung cancer (LUAD, LUSC; KRAS-driven)	IRF3, TBK1, and IFI measured using immunoblot	↓ STING/↓ cGAS (compared to normal lung)	Lower expression → poorer outcomes, stage-dependent	Reduced immune infiltration in advanced disease; STING higher in PBMCs of localized disease	Stage-dependent; localized disease may benefit from STING activation	Promoter hypermethylation; LKB1 loss and SAMHD1 overexpression linked to STING suppression	(170–176)
Colorectal cancer (MSI-H/POLE-mutant)	cGAS and STING protein levels quantified using IHC	↑ STING activation in MSI-H/POLE	Active STING → better survival, earlier stage detection	Inflamed TME, ↑ CD8^+^ CTLs, chemokine/IFN signatures	Strong response to ICIs in MSI-H/POLE tumors	High TMB/neoantigen load drives cGAS–STING; suppressed STING associated with ECM-rich, immunosuppressive stroma	(177–180)
Endometrial cancer (including POLE-mutant)	mRNA of IFN and cGAS with RT-PCR	Generally ↓ (ERα-mediated repression); ↑ in POLE-mutant	STING-active (POLE) cases show favorable outcomes	POLE-mutant: inflamed TME and IFN signatures	Potential responsiveness to STING agonists/ICIs in POLE-mutant cases	ERα recruits HDAC3 to STING promoter → epigenetic silencing; POLE mutations increase cytosolic DNA	(103, 181)
Breast—triple negative (TNBC)	Protein level of STING, TBK1, and STAT1 detected by IHC	↑ STING signature (subtype-specific)	Often better PFS, but dual roles observed	↑ Genomic instability, ↑ immune infiltration; SASP/IL-6 signaling possible	Some TNBCs may respond to ICIs; context-dependent	STING → IL-6 → STAT3 can promote survival under genotoxic stress; chronic activation may support tumor growth	(42, 83, 182)
Breast—HER2^+^	TBK1 detected with Western blotting, IRF3 with immunofluorescence	Variable; ↓ in trastuzumab-resistant tumors	Low STING associated with poor prognosis and therapy resistance	Immune-evasive TME with reduced antigen presentation	Lower pCR rates in low-STING; combination strategies may help	AKT1 impairs TBK1/IRF3/STING assembly; downregulation facilitates immune escape	(183, 184)
Breast—luminal (ER+/PR+)	cGAS, TBK1, STING, and IRF3 quantified using Western blotting	Often ↓ in endocrine-resistant tumors	Reduced STING linked to endocrine resistance	Lower immune activation: macrophage infiltration noted	May predict resistance to endocrine therapy	AKT1-mediated inhibition of STING complex assembly implicated	(184, 185)
Cervical and HPV+ oropharyngeal cancers	*In silico* analysis of STING downstream genes (CCL5, CXCL7, CXCL10)	↓ STING due to viral proteins (E6/E7)	Suppressed STING → worse outcomes/increased recurrence	Immune-cold phenotype despite viral DNA	Rationale for STING agonists ± viral protein or TLR inhibitors	HPV E7 binds/inhibits STING; E6/E7 epigenetically suppress interferon genes	(186–190)
Head and neck SCC (HPV−)	*In silico* analysis of cGAS level and quantification using Western blotting	↑ STING expression in many cases	Associated with improved OS/PFS/DFS	↑ TILs, NK cells; also increased immunosuppressive cells (Tregs/MDSCs) in some contexts	Better chemo- and immunotherapy responses in STING-high patients	STING may recruit both anti- and pro-tumor immune subsets; context-dependent	(191–193)
Head and neck SCC (HPV+)	cGAS and STING quantified with Western blotting	↓ STING (viral suppression)	Worse prognosis vs STING-active cases	Immune evasion via viral mechanisms	May need combined strategies to overcome viral suppression	HPV16/18 E7 inhibits STING signaling via NLRX1 engagement and an LXCXE-dependent mechanism, promoting STING turnover and suppression of type I interferon responses.	(188, 194, 195)
Pancreatic ductal adenocarcinoma (PDAC)	STING and TBK1 protein levels quantified with immunofluorescence	↓ cGAS/STING (low expression)	Low STING correlates with poor prognosis	Desmoplastic, immune-excluded (“cold”) TME	Poor response to ICIs; STING agonists under investigation	TF/TREX1 axis and DTX3L-mediated cGAS degradation suppress STING signaling	(168, 196, 197)
Bladder (urothelial) carcinoma	*In silico* analysis of STING and SASP	Variable (context/subtype dependent)	↑ STING often linked to favorable immunotherapy response	Immune effector infiltration in low risk; macrophage predominance in high-risk	Higher TMB/MSI associated with better ICI response	SRC proto-oncogene may inhibit cGAS activity; nomogram/risk models proposed	(198–201)
Renal cell carcinoma (KIRC, KIRP)	cGAS and IRF3 quantified using Western blotting	↑ STING/cGAS signatures frequent	Paradox: ↑ STING with ↑ immune infiltration but worse prognosis	↑ CD8^+^/CD4^+^ T cells and features of immune activation alongside aggressive behavior	Mixed ICI responsiveness; complex biology	Aneuploidy and loss of SETD2/PBRM1 increase cytosolic DNA → STING activation that may reflect genomic instability	(191, 202–205)
High-grade serous ovarian cancer (HGSOC)	IRF3, STING, and TBK1 protein levels quantified with Western blotting	↑ STING in BRCA/HRD tumors	STING-active tumors show better PFS/OS	Immune-hot TME in STING-active cases	Potentially improved response to ICIs and combination therapies	HRD/BRCA mutations and loss of DNA repair (e.g., TP53BP1) → micronuclei → STING activation; epigenetic silencing can occur	(206–210)
Cutaneous squamous cell carcinoma (cSCC)	*In silico* analysis of cGAS expression level	Variable; often similar to normal skin	Higher cGAS linked to improved OS in some analyses	Mixed immune features; promoter hypomethylation noted without major expression shifts	Limited but suggestive data for immunotherapy benefit	TCGA analyses show no significant tumor vs normal difference for cGAS in some datasets	(168, 198)
Merkel cell carcinoma (MCC)	*In silico* analysis of cGAS and STING. Further quantification with Western blotting and immunofluorescence	Often silenced in tumor cells despite viral/UV association	Silencing contributes to immunologically “cold” TME and poorer outcomes	Immune and stromal cells can show high STING expression	Potential target for STING activation strategies in subsets	Virus-driven or epigenetic silencing reduces tumor-intrinsic STING	(211–213)
Pheochromocytoma/paraganglioma (PCPG)	*In silico* analysis of cGAS downstream genes (CCL3, CCL4)	↑ STING in immune/stromal compartments	Favorable immune profile when STING-active	Inflamed stromal and immune microenvironment in some cases	Potential sensitivity to immunotherapy in select cases	High STING in non-tumor compartments may drive local immune activity	(211, 214)
Prostate cancer	TBK1, cGAS, and STING quantified with Western blotting	Variable; tumor-intrinsic STING required for some responses	STING-sufficient tumors show slower growth and better immune control	↑ Leukocytes and DCs with STING activity	STING agonists can suppress growth in STING-expressing tumors	Tumor-intrinsic STING required for cGAMP-mediated antitumor effects in models	(145)
Glioblastoma (GBM)	IRF3, TBK1, cGAS, and STING quantified with Western blotting	Tumor cell STING often epigenetically silenced; stromal/myeloid expression retained	Silencing linked to immune evasion, poor prognosis	Myeloid and vascular STING expression can trigger local cytokine release	Preclinical models: intracranial STING agonists remodel TME and prolong survival	Promoter hypermethylation of STING; decitabine can restore expression in cell lines	(149–153)
Acute myeloid leukemia (AML)	*In silico* analysis of STING genes	↑ cGAS/STING expression (mRNA)	Higher expression correlates with poorer overall and disease-free survival	Pro-inflammatory milieu: chronic activation may promote progression	Limited ICI benefit; potential distinct therapeutic implications	Chronic low-grade STING activation may create pro-tumor inflammation	(215, 216)
Diffuse large B-cell lymphoma (DLBCL)	*In silico* analysis of STING genes	↑ STING pathway activity in subsets	Associated with better prognosis in some studies	Tumor immune activation signatures present	May be responsive to immune-modulatory strategies	STING upregulation contributes to antitumor immunity in DLBCL context	(216)
Multiple myeloma (MM)	*In silico* analysis of STING genes	↑ STING/cGAS in some analyses	Associated with poorer outcomes	Pro-inflammatory and tumor-promoting microenvironment features	Immunotherapy utility unclear; more study needed	Upregulation linked to disease progression in datasets	(216)
Peripheral T-cell lymphoma (PTCL)	*In silico* analysis of STING genes. Further confirmation of cGAS and STING protein expression with Western blotting and immunofluorescence	↑ cGAS–STING by single-cell RNA-seq	Elevated expression correlates with proliferation and worse outcomes	High proliferative index drives tumor progression	Not an ICI-favored context; targeted inhibition showed tumor regression in models	cGAS may act as oncogene in PTCL; inhibition leads to tumor shrinkage	(217)

NSCLC, non-small cell lung cancer; TNBC, triple-negative breast cancer; PDAC, pancreatic ductal adenocarcinoma; HNSCC, head and neck squamous cell carcinoma; HGSOC, high-grade serous ovarian cancer; cSCC, cutaneous squamous cell carcinoma; RCC, renal cell carcinoma; SKCM, skin cutaneous melanoma; TILs, tumor-infiltrating lymphocytes; ICIs, immune checkpoint inhibitors; BRAF, B-Rapidly Accelerated Fibrosarcoma; cGAS, cyclic GMP-AMP synthase; STING, stimulator of interferon genes; LUAD, lung adenocarcinoma; LUSC, lung squamous cell carcinoma; KRAS, Kirsten rat sarcoma; PBMCs, peripheral blood mononuclear cells; LKB1, liver kinase B1; SAMHD1, SAM and HD domain-containing deoxynucleoside triphosphate triphosphohydrolase-1; MSI-H, microsatelite-instability high; POLE-mutant, DNA polymerase epsilon mutant; CTLs, cytotoxic T lymphocytes; TMB, tumor mutational burden; ECM, extracellular matrix; TME, tumor microenvironment; IFN, interferon; HDAC3, histone deacetylase-3; PFS, progression-free survival; TNBCs, triple-negative breast cancer; IL-6, interleukin-6; STAT3, signal transducer and activator of transcription 3; ERα, estrogen-receptor alpha; SASP, senescence-associated secretory phenotype; HER2^+^, human epidermal growth factor positive; TBK1, TANK-binding kinase-1; IRF3, interferon regulatory factor-3; ER+/PR+, estrogen+/progesterone+; TLR, Toll-like receptor; NLRX1, NLR family member X1; LXCXE, Leu-X-Cys-X-Glu; TREX1, Three Prime Repair Exonuclease 1; DTX3L, Deltex E3 Ubiquitin Ligase 3-like; SRC, sarcoma; HRD, homologous recombination deficiency; OS, overall survival; BRCA, breast cancer; TCGA, The Cancer Genome Atlas; UV, ultraviolet; DCs, dendritic cells; RNA-seq, ribonucleic acid sequencing.

The interpretation of STING pathway-associated biomarkers is highly dependent on both the detection modality and the biological source of the sample analyzed. While several experimental and translational studies have successfully measured phosphorylated signaling intermediates like TBK1, IRF3, and cGAMP, these readouts typically require fresh or optimally preserved tumor tissue, specialized reagents, and highly controlled pre-analytical conditions, limiting their routine applicability in clinical practice. In contrast, transcriptomic approaches (e.g., TCGA-based RNA sequencing or RT-PCR) and protein-level assessment using immunohistochemistry or immunofluorescence are more broadly feasible but primarily capture pathway component abundance or downstream-driven programs rather than real-time signaling dynamics. Sample source further influences interpretability, as clinical biopsies, such as bulk tumor tissues, integrate signals from malignant tumors, stromal, and immune components, whereas peripheral blood-based measurements preferentially reflect systemic interferon responses and may not accurately recapitulate intratumoral STING activity. Consequently, current clinical correlation between STING pathway activation and immunotherapy response often relies on surrogate or composite biomarkers whose predictive value is shaped by assay sensitivity, tissue context, and the timing of sample acquisition, underscoring the need for standardized, clinically tractable assays that capture functionally relevant STING signaling states.

## 7 Strategies for synergistic potential of STING agonism in cancer therapy

The cGAS–STING pathway has emerged as a pivotal mediator of innate immune sensing of cytosolic DNA, with significant implications for cancer therapy. While STING agonism alone has demonstrated substantial antitumor effects, mounting evidence suggests that its greatest translational promise lies in combination strategies with established cancer treatments, such as chemotherapy, radiotherapy, and immunotherapy. These combinations exploit the complementary mechanisms of action to enhance tumor immunogenicity, overcome immune resistance, and ultimately improve clinical outcomes.

In this section, we examine how standard-of-care therapies such as chemotherapy and radiotherapy can synergize with natural or synthetic STING ligands to activate the cGAS–STING pathway, thereby transforming the TME into a more immunostimulatory niche. Similarly, immunotherapies such as ICIs, cancer vaccines, and chimeric antigen receptor (CAR) T cells can be potentiated by the pro-inflammatory milieu generated through STING activation. This synergistic interplay provides a compelling rationale for integrated therapeutic approaches that integrate STING agonism to boost antitumor immunity. Subsequently, we provide the mechanistic underpinnings and preclinical evidence supporting each combination modality.

### 7.1 STING agonism and radiotherapy

RT induces extensive DNA double-strand breaks (DSBs) that generate profound cytosolic DNA fragments, micronuclei, and other DNA structures capable of activating the cGAS–STING pathway. While RT can trigger the cGAS–STING pathway in tumor or immune cells, this effect is often not durable, necessitating the application of STING agonists in synergy (218). However, this approach currently remains in the early stages of clinical exploration, with only a limited number of trials initiated.

Preclinical evidence suggests a dual immune response to RT, which is likely dependent on radiation dose. In murine models, treatment of CT26 colorectal cancer or 4T1 breast cancer cells with radiation has been demonstrated to initiate effective STING-dependent CTL responses (219). Furthermore, cGAMP treatment has been shown to augment the antitumor effects of radiation in a hepatocellular carcinoma mouse model, thereby bolstering the cytotoxic capabilities of tumor-specific CD8^+^ T cells (220). RT also enhances the expression of MHC class I molecules on tumor cells, thereby facilitating the activation of tumor-specific T lymphocyte responses. The IFN-β, downstream of STING activation, has been shown to induce the expression of captivating receptor (NKG2D) on NK cells, which interacts with UL16-binding protein (ULBP1-6) and MHC class I chain-related proteins A and B (MICA/B) on tumor cells, enhancing the direct cytotoxicity of NK cells (221).

Additional preclinical models demonstrate how the rational combination of RT with STING agonists can overcome these suppressive effects. In one model, the intratumoral delivery of the STING agonist, R_P_, and R_p_ ditho c-di-GMP (RR-CDG) following RT preferentially activated STING in tumor stromal cells, particularly tumor-associated macrophages, triggering rapid NF-κB-dependent TNFα and CCL2 production. This acute innate response induced dose-dependent vascular disruption and hemorrhagic tumor necrosis, counteracting radiation-induced macrophage immunosuppressive polarization. In parallel, STING-driven type I interferon signaling permits dendritic cells to cross-present radiation-released tumor antigens, enabling robust CD8^+^ T-cell priming and durable local and metastatic tumor control in the PDAC mouse model (222). Complementary evidence comes from lung metastasis models in which aerosolized phosphatidylserine-coated nanoparticles delivering cGAMP (NP-cGAMP) selectively targeted alveolar macrophages and CD103^+^ dendritic cells. When combined with fractionated lung irradiation (8 Gy × 3), NP-cGAMP amplified STING–TBK1–IRF3 signaling in intratumoral APCs, enhanced the cross-presentation of radiation-released antigens, and drove the massive expansion of tumor-specific CD8^+^ T cells. This combination converted localized RT into a systemic, CD8^+^ T cell-dependent antitumor response capable of inducing abscopal regression of distant lung metastases (223).

Collectively, these findings highlight the promise of the STING pathway as a potential strategy for inducing potent antitumor immune responses. Nonetheless, the positive effects of RT on antigen presentation, DC function, CD8^+^ T-cell infiltration, and NK cell activity are counterbalanced by inhibitory signals induced by RT, dampening this beneficial immune activation. For instance, high single-fraction doses of RT (typically above 12–18 Gy) induce a cytoplasmic exonuclease called TREX1 (224), which degrades cytosolic DNA fragments and prevents cGAS activation, effectively suppressing the type I interferon response. Additionally, RT upregulates 3-hydroxy-3-methylglutaryl-CoA reductase (HMGCR), a rate-limiting enzyme in cholesterol biosynthesis (225). The resulting elevated cholesterol levels in tumor cells trap STING in the endoplasmic reticulum, preventing its activation and blocking downstream interferon signaling. This inhibition of the cGAS–STING pathway suppresses the immune response, allowing tumors to evade immune destruction and resist RT in the colorectal cancer model. Clinically, higher HMGCR and cholesterol levels in CRC patients correlate with worse prognosis and poorer responses to RT. Importantly, pharmacologic inhibition of HMGCR restores cGAS–STING activity and significantly improves RT response in animal models via enhanced antitumor immunity. This suggests that combining statins with radiotherapy offers a promising strategy to overcome resistance by blocking the cholesterol-mediated suppression of cGAS–STING signaling, thereby improving treatment outcomes in colorectal cancer. Furthermore, radiation generates significant oxidative stress, which can activate the nuclear factor erythroid 2-related factor 2 (NRF2) pathway. NRF2, a key transcription factor involved in oxidative stress response, has been shown to attenuate cGAS–STING signaling (226), although the precise molecular mechanisms of this suppression require further elucidation. Together, while RT can activate the cGAS–STING pathway and synergize with STING agonist to promote antitumor immunity, higher radiation dose impairs this pathway, contributing to therapy resistance. Therefore, to optimize therapeutic outcomes, it is critical to balance these opposing effects on the cGAS–STING pathway. This can be through fractionated radiation schedules with moderate doses per fraction or by combining RT with pharmacologic inhibitors targeting TREX1, cholesterol biosynthesis, or NRF2 pathways. These approaches may present promising strategies to relieve these negative regulatory constraints and amplify tumor immunogenicity.

### 7.2 STING agonism and chemotherapy

Similar to RT, several chemotherapeutic agents, including cisplatin, gemcitabine, paclitaxel, etoposide, and topoisomerase inhibitors, indirectly activate the cGAS–STING axis by generating cytosolic DNA fragments. For instance, cisplatin and gemcitabine activate the STING-dependent interferon pathway in nasopharyngeal carcinoma (227), leading to increased MHC class I expression, elevated IL-1β production, and the expansion of antitumor CD8^+^ T cells. In ovarian and bladder cancers, cisplatin-activated cGAS–STING signaling not only suppresses tumor cell proliferation but also enhances infiltration of CD8^+^ T cells and dendritic cells (139, 228). Paclitaxel, a microtubule-targeting agent, stimulates STING-mediated pro-inflammatory responses by activating STING, inducing interferon signaling, and triggering downstream immune effects (229). Similarly, topoisomerase inhibitors such as teniposide activate the cGAS–STING pathway (230), thereby strengthening antitumor T-cell responses. Mechanistically, this activation triggers the production of type I interferons and other inflammatory signals, enhancing dendritic cell function and promoting the infiltration and activation of cytotoxic CD8^+^ T cells in the TME. Importantly, experimental evidence shows that the knockdown of STING impairs teniposide’s antitumor efficacy and its ability to stimulate immune cells, confirming that the antitumor immune response induced by teniposide critically depends on tumor-intrinsic STING activation. In summary, these findings collectively underscore the critical role of the cGAS–STING pathway in the context of chemotherapy for cancer treatment.

### 7.3 STING agonism and immunotherapy

The cGAS–STING pathway has emerged as a central immune regulator with the capacity to reshape the TME, enhance dendritic cell-mediated T-cell priming, and sustain long-term antitumor memory (231). While STING agonists alone can induce measurable tumor regression, their efficacy is often limited by adaptive resistance mechanisms characterized by checkpoint upregulation and T-cell exhaustion in the long term. These limitations have spurred intense interest in combining STING activation with other immunotherapies. Preclinical and early clinical studies have suggested that STING agonism can sensitize tumors to immune checkpoint inhibitors, augment CAR T-cell infiltration and persistence, and boost the potency of cancer vaccines by acting as a molecular adjuvant.

### 7.4 Immune checkpoints and STING agonists in cancer immunotherapy

The upregulation of immune checkpoint molecules, notably PD-L1 on tumor cells and PD-1 on T cells, is a hallmark of chronic immune activation and a significant mechanism of immune suppression within the TME. PD-1 engagement by tumor-expressed PD-L1 delivers inhibitory signals that suppress T-cell activation and cytotoxicity, enabling tumor immune evasion. Importantly, the chronic activation of the STING pathway has been linked to the upregulation of these checkpoint molecules, a phenomenon documented across multiple cancers, including hepatocellular carcinoma, melanoma, and lung cancers (220, 232–235). This has prompted the therapeutic rationale that combining STING activation with immune checkpoint blockade could overcome tumor resistance and improve antitumor immunity.

Preclinical studies have supported this approach, showing that STING agonists significantly enhance antitumor effects when combined with PD-1 or PD-L1 inhibitors (127, 236, 237). These combinations boost T-cell activation and infiltration, leading to durable tumor control. The efficacy of checkpoint blockade is closely tied to STING pathway integrity. In melanoma models, it was shown that radiation combined with anti-CTLA-4 therapy promoted tumor elimination only when tumors retained functional STING signaling, as STING-deficient tumors exhibited impaired CD8^+^ T-cell infiltration and diminished therapeutic benefit (238). These findings highlight STING as a prerequisite for optimal CTLA-4 blockade. In addition to CTLA-4, various researchers have shown that the antitumor effects of STING agonists are significantly enhanced when administered in combination with PD-1 or PD-L1 inhibitors (127, 236, 237), resulting in improved T-cell activation, infiltration, and durable tumor control.

Extending these observations, additional preclinical studies have demonstrated that STING agonists can convert immunologically “cold” peritoneal tumors into checkpoint-responsive lesions when appropriately combined with immune checkpoint blockade. In a syngeneic peritoneal carcinomatosis of colon cancer (PCCC) model generated by intraperitoneal implantation of MC38 cells into C57BL/6 mice, the chronic activation of STING with the synthetic agonist RR-CDA (MIW815/ADU-S100) rapidly induced type I interferon signaling in intratumoral dendritic cells, leading to vascular normalization, macrophage repolarization toward an M1 phenotype, and restoration of CD8^+^ T-cell infiltration within 10–18 days. Although STING monotherapy partially suppressed tumor burden, it concurrently induced adaptive immune resistance via PD-L1 upregulation on myeloid cells. This limitation was overcome by anti-PD-1 therapy, resulting in robust CD8^+^ T-cell activation, increased IFN-γ production, normalized tumor vasculature, and near-complete eradication of peritoneal tumors and malignant ascites in a substantial fraction of treated mice. Therapeutic synergy required intact type I IFN signaling and CD8^+^ T cells, as IFNAR blockade or CD8 depletion abrogated both immune and vascular remodeling (143).

A similar immunologic reprogramming has been observed in ovarian cancer. In the ID8-Trp53−/− syngeneic model of HGSOC with peritoneal dissemination, the intraperitoneal administration of a synthetic STING agonist significantly prolonged survival, reduced malignant ascites, and decreased tumor burden without direct tumor cytotoxicity. STING activation induced a type I interferon-driven transcriptional program characterized by enhanced antigen presentation, STAT1–CXCL10 signaling, and the accumulation of activated CD103^+^ dendritic cells, thereby promoting CD8^+^ T-cell recruitment and cross-priming. However, adaptive immune resistance emerged at intermediate time points, marked by PD-1 upregulation on CD8^+^ T cells and PD-L1 induction on myeloid populations. The sequential administration of carboplatin followed by STING agonist sensitized tumors to immune checkpoint blockade, and triple combination therapy (carboplatin, STING agonist, and anti-PD-1) produced the greatest survival benefit without added toxicity. Persistent STING-driven cytokine responses (CXCL9/10, IFN-γ, and CCL5), partially independent of CD8^+^ T cells, suggested additional contributions from NK cells and myeloid compartments (239). Together, these studies have demonstrated that STING agonism not only enhances the efficacy of ICI but also expands the diversity and potency of antitumor T-cell responses, offering a compelling rationale for combinatorial strategies in the clinic.

Clinically, early-phase trials have produced mixed results, with inconsistent activity observed for STING agonist and immune checkpoint inhibitor combinations. For example, in a multicenter Phase 2 trial (NCT03937141), the synthetic STING agonist ADU-S100 combined with pembrolizumab in patients with recurrent or metastatic head and neck cancer was prematurely terminated due to a lack of significant antitumor efficacy. In contrast, a Phase 1 trial (NCT03991559) combining manganese (Mn^2+^), a cGAS activator, with anti-PD-1 therapy showed an objective response rate of 45.5% and disease control in 90.9% of patients with advanced metastatic solid tumors, including those previously refractory to PD-1 therapy. These findings, however, highlight the translational potential of leveraging STING agonists, while also revealing a clinical gap that needs to be addressed for STING agonists to reach their full potential.

### 7.5 Combining STING agonists with chimeric antigen receptor T-cell therapy

CAR T-cell therapy is one of the most promising anti-cancer strategies and has shown remarkable efficacy in hematologic malignancies, yet its success in solid tumors remains limited. This discrepancy is largely attributed to the highly immunosuppressive TME and intratumoral heterogeneity, which impair CAR T-cell infiltration, persistence, and function (240). To address this barrier, recent studies have explored STING agonists as a means to “heat” the TME and thereby enhance CAR T-cell antitumor activity.

In a syngeneic mouse mammary tumor model, the intratumoral or peritumoral administration of STING agonists (DMXAA and cGAMP) significantly boosted the efficacy of CAR T cells. Mechanistically, this effect was linked to the upregulation of T cell-homing signals CXCL9 and CXCL10, promoting CAR T-cell infiltration into tumor tissue. However, sustained tumor regression required combination with anti-PD-1 antibodies, likely due to the reversal of CAR T-cell exhaustion (241). Consistent with this, Feng Ji et al. reported that poly(ADP-ribose) polymerase inhibitors (PARPi) can activate the cGAS–STING pathway and thereby enhance the antitumor efficacy of CD70-directed CAR T cells in renal cancer (242). More recently, a novel strategy using anti-PD-L1 nanovesicles loaded with cGAMP (aPD-L1 NVs@cGAMP) has been developed to improve the efficacy of CAR T-cell therapy. This formulation delivered STING agonists directly to lung tumors, inducing IFN-I responses, recruiting pro-inflammatory immune cells, and, critically, blocking PD-L1 upregulation that would otherwise exhaust CAR T cells. In a metastatic lung cancer model, CAR T cells combined with aPD-L1 NVs@cGAMP eradicated established tumors, prevented recurrence upon rechallenge, and avoided systemic inflammation (243).

The co-delivery of STING agonists with CAR T cells has also been shown to enhance systemic tumor-specific T-cell function. In a pancreatic tumor model, the combination of cyclic dinucleotide (cdGMP) and CAR T cells significantly enhanced TCR signaling in tumor-specific T cells, leading to complete tumor clearance and durable protection upon rechallenge, indicating the induction of long-term systemic immunity (244). Parallel efforts have applied this principle to CD70 CAR T cells, where PARPi-mediated STING activation reprograms the TME, enabling even low-dose CAR T-cell therapy to drive effective tumor regression (242). Moreover, new-generation STING agonist IMSA101 stimulates IL-18 secretion and further enhances CAR T-cell efficacy (245). Collectively, these studies have highlighted a growing body of evidence that strategically combining STING pathway activation with CAR T-cell therapy can overcome the immunosuppressive TME, enhance T-cell persistence, and induce durable systemic immunity. This synergy holds significant promise for translating CAR T-cell therapy into effective treatments for solid tumors.

An emerging strategy to decouple beneficial targeted STING activation from detrimental T cell-intrinsic effects is to combine STING agonists with engineered T cells that lack STING signaling. A recent study assessed CRISPR-edited STING-ablated CAR T-cell combined with the STING agonist (diABZI) in pancreatic cancer models. This resulted in enhanced tumor killing, increased CAR T-cell proliferation, reduced exhaustion, and the expansion of effector-memory T-cell phenotype (246). Mechanistically, to achieve such benefits, STING ablation in CAR T cells is required while relying on tumor-intrinsic STING signaling during STING treatment, providing a concrete translational rationale for pairing STING agonists with STING-deficient adoptive therapies in immune-deprived solid tumors.

### 7.6 STING agonist as an adjuvant for cancer vaccines

A critical challenge in cancer vaccine development is overcoming immune tolerance and eliciting robust tumor-specific immunity (247). Selecting appropriate adjuvants is essential, as they activate innate immunity and stimulate APCs, thereby enhancing the immunogenicity of tumor-associated antigens (TAAs) (248). The prevailing evidence shows that adjuvants can amplify pre-existing TAA-specific responses and promote the development of new tumor-specific immunity. Given its pivotal role in initiating innate immune responses, the STING pathway has emerged as a promising target for cancer vaccine adjuvants (247).

Fu et al. developed one of the earliest generations, called STINGVAX, a cancer vaccine formulated by combining cyclic dinucleotides (CDNs) as STING agonists with granulocyte-macrophage colony-stimulating factor-producing cancer cells to enhance dendritic cell activation and tumor antigen-specific CD8^+^ T-cell responses (249). In multiple murine models, including melanoma, colon carcinoma, upper aero-digestive squamous cell carcinoma, and pancreatic carcinoma, STINGVAX demonstrated robust antitumor efficacy compared to GM-CSF alone (249). This broad-spectrum antitumor activity indicates the potential for STINGVAX to generate potent, durable immune activation across diverse tumor types. Importantly, synthetic CDNs were shown to potently activate both mouse and human dendritic cells, supporting the translational potential of STING-based vaccine strategies.

**Figure 3:**
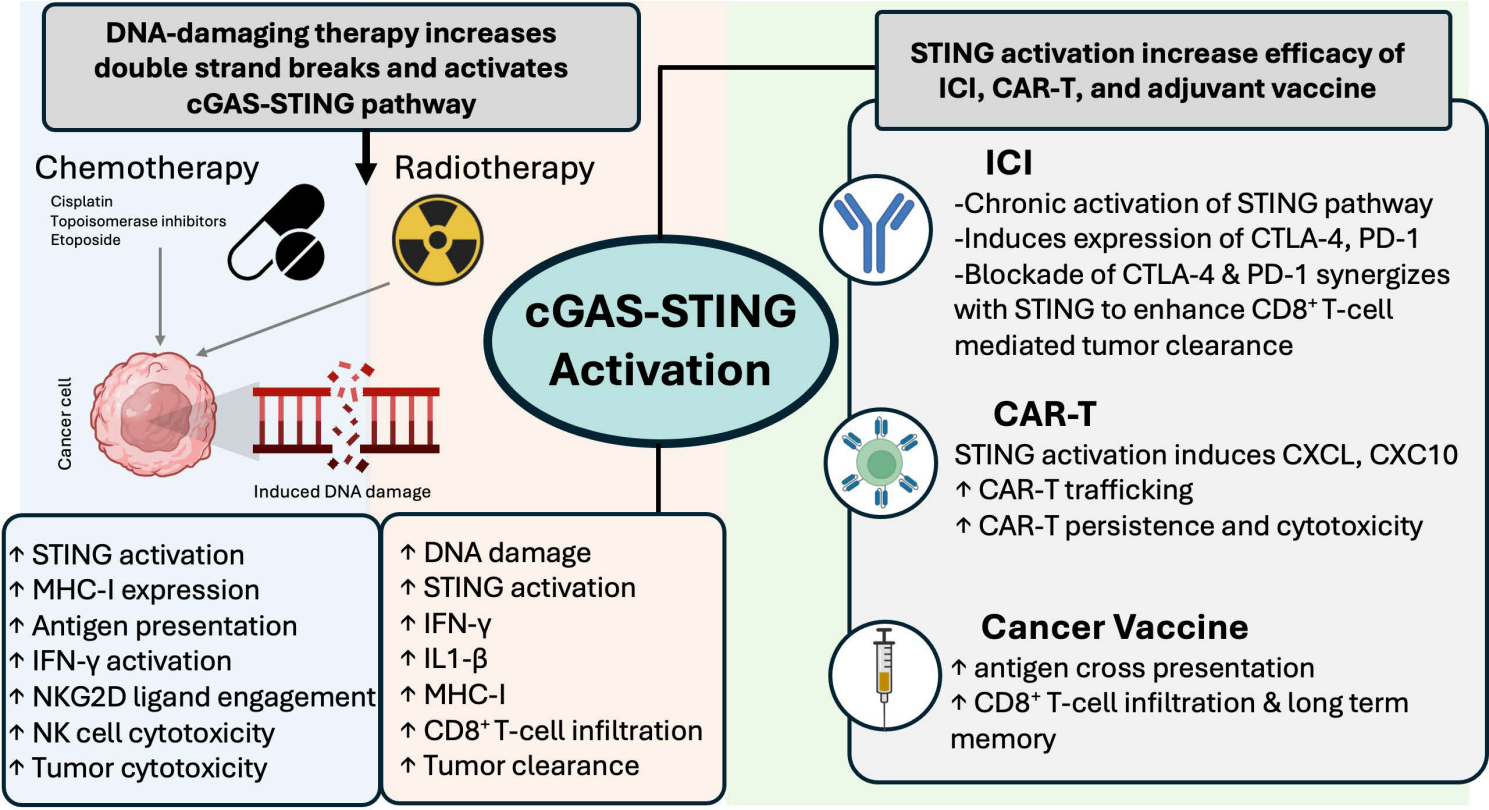
Strategies for synergistic potential of STING agonism. DNA-damaging therapies, including chemotherapy (e.g., cisplatin, topoisomerase inhibitors, and etoposide) and radiotherapy, induce double-strand DNA breaks, leading to activation of the cGAS-STING signaling pathways in cancer cells. This activation results in increased MHC-I expression, antigen presentation, IFN-γ signaling, NK-cell cytotoxicity, and CD8+ T-cell infiltration, ultimately promoting tumor cytotoxicity and clearance. Activation of cGAS-STING pathways also potentiates the increased efficacy of immune checkpoint inhibitors, CAR-T cell therapy, and cancer adjuvant vaccines. cGAS, cyclin GMP-AMP synthase; STING, stimulator of interferon genes; ICI, immune checkpoint inhibitor; CAR-T, chimeric antigen receptor T-cell therapy.

In breast cancer, vaccine development has long been limited by the lack of highly immunogenic TAAs. Recent studies, however, have shown that STING agonists can serve as potent adjuvants to enhance vaccine-induced antitumor immunity. When HSP90-derived peptide vaccines targeting HER2^+^ cells were combined with STING agonists, they markedly enhanced antitumor immunity in MMTVneu-transgenic mice (250). This STING activation boosts systemic HSP90-specific T-cell responses, with increased T-cell infiltration into the TME, intermolecular epitope spreading, and expanded TCRβ diversity, thereby amplifying both CD4^+^ and cross-primed CD8^+^ T-cell responses (250). This combination led to greater tumor rejection compared to monotherapy, demonstrating the benefit of STING agonists as potent adjuvants to improve the efficacy of cancer vaccines. Collectively, these studies have demonstrated that STING agonists can act as potent vaccine adjuvants, enhancing antigen-specific cellular immunity, promoting tumor antigen presentation, and providing broad antitumor protection. **Figure 3** fully captures relevant synergistic approaches that can be devised in the development of STING agonists.

## 8 Clinical translation and therapeutic strategies

The translation of STING agonists into clinical settings has been recently accelerated, with multiple early-phase trials assessing their safety, pharmacodynamics, and preliminary efficacy in solid tumors and lymphomas ((251); NCT01762020, NCT05465161, NCT05549804, and NCT05387928). Oftentimes, due to their combinatorial potential, these agents are combined with checkpoint inhibitors such as PD-1, PD-L1, and CTLA-4 antibodies, or chemotherapy to enhance antitumor immunity, particularly in immunologically “cold” tumors. Several classes of STING agonists are being explored to leverage this innate sensing mechanism for therapeutic benefit in cancer. Some of these agents and their mechanisms of action are briefly discussed subsequently.

### 8.1 Cyclic dinucleotide-based STING agonists

The first class includes CDN-based STING agonists, which mimic natural cGAMP and bind directly to STING. One of the most extensively studied is ADU-S100 (MIW815), a synthetic CDN developed by Novartis (formerly Aduro Biotech) (252). Delivered intratumorally, ADU-S100 has been tested in patients with advanced or metastatic solid tumors and lymphomas ((253); NCT02675439 and NCT03172936). Although well-tolerated, ADU-S100 demonstrated limited monotherapy activity, as described in **Table 2**; systemic immune activation was claimed (252). However, its combination with spartalizumab (anti-PD-1) showed modest responses in adult patients with PD-L1+ recurrent or metastatic head and neck squamous cell carcinoma (HNSCC) (254) as well as in advanced metastatic tumors or lymphoma (255). Despite this, overall tumor control remains limited, leading Novartis to discontinue development.

**Table 2:** Selected clinical trials targeting the cGAS–STING pathway.

Agonist	Molecular type	Dosing route	Indication	Sponsor	Clinical phase	NCT number	Results	Reference
MIW815 (ADU-S100)	CDN analog	Intratumoral	Advanced/metastatic solid tumors and lymphomas	Novartis AG	I/II	NCT02675439	Limited efficacy as a monotherapy, well-tolerated.	(252)
(MIW815) and spartalizumab	CDN analog	Intratumoral + IV	Advanced/metastatic solid tumors or lymphomas	N/A	Ib	NCT03172936	MIW815 + spartalizumab combination had a manageable safety profile. Minimal antitumor responses were seen (overall response rate was 10.4%).	(255)
MK-1454	Synthetic CDN	IT	Advanced solid tumors and lymphomas; metastatic or unresectable recurrent HNSCC	Merck Sharp & Dohme LLC	I/II, II	Phase I: NCT03010176 Phase II: NCT04220866	Phase I: Pyrexia (70%); dose-dependent PK; transient CXCL10/IFN-γ/IL-6 induction without dose–response beyond 540 µg. ORR 50% (4/8) with combination vs 10% (1/10) with pembrolizumab; pyrexia most common AE.	(256, 262)
Mnl2 + sintilimab	NA	Inhalation	Platinum-resistant/refractory ovarian cancer	NA	2	NCT03989336	78.6% PRs and 100% disease control, without substantial added toxicity.	(263)
ONM-501 + cemiplimab	Dual-targeted agonist	IT	Advanced solid tumors/lymphomas	NA	I	NCT06022029	First-in-human Phase I trial started in November 2023; testing ONM-501 intratumorally alone and with anti-PD-1 cemiplimab in advanced solid tumors/lymphomas.	(264)
CRD3874-SI	Small molecule	IV	Advanced sarcoma and Merkel cell carcinoma	Curadev Pharma, Inc.	I	NCT06021626	Still in early stages of dose escalation.	(265)
SB11285	Small molecule	IV	Advanced solid tumors	Spring Bank Pharmaceuticals, Inc.	I/II	NCT04096638	Still in early stages of dose escalation.	(266)
BMS-986301	CDN analog	IT, IV	Advanced solid cancers	Bristol-Myers Squibb Co.	I	NCT03956680	Still in early stages of dose escalation.	
E7766	Macrocycle-bridged agonist	IT	Advanced solid tumors and lymphomas, non-muscle invasive bladder cancer	Eisai Inc.	I, I/Ib	NCT04144140 NCT04109092	NCT04144140 (E7766; Phase I/1b): trial terminated; no evidence of clinical efficacy reported to date.	(266, 267)
GSK3745417	Non-CDN	IV	Relapsed/refractory solid tumors	GlaxoSmithKline	I	NCT03843359	NCT03843359 (GSK3745417; Phase I): active, not recruiting; no evidence of clinical efficacy reported.	
MK-2118	Synthetic CDN	IT	Advanced/metastatic solid tumors or lymphomas	Merck Sharp & Dohme LLC	I	NCT03249792	NCT03249792 (MK-2118; Phase I): completed/terminated; no evidence of clinical efficacy reported.	(268)
XMT-2056	ADC	IV	Advanced/metastatic HER2^+^ Solid Tumors	Mersana Therapeutics	I	NCT05514717	Early-phase; efficacy not yet demonstrated.	

CDN, cyclic dinucleotide; IV, intravenous; IT, intratumoral; ADC, antibody-drug conjugate; HER2^+^, human epidermal growth factor receptor-2.

**Figure 4:**
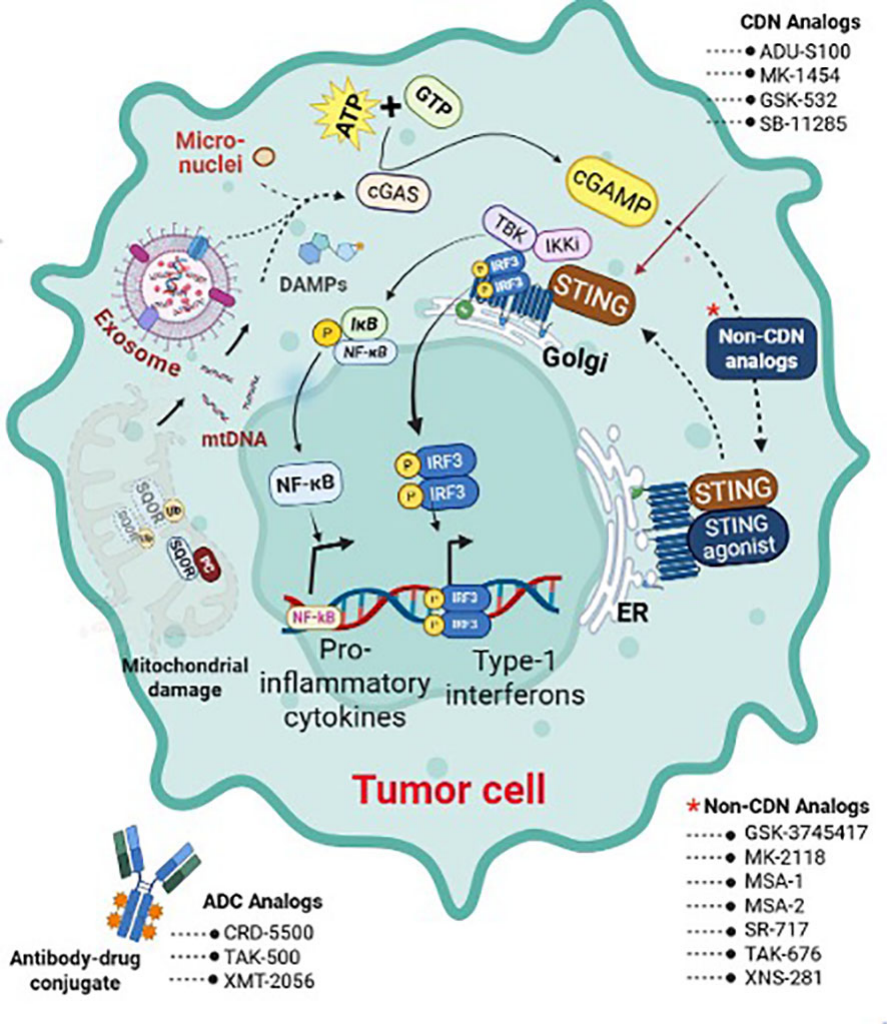
Overview of cGAS–STING signaling activation in tumor cells and the classes of STING-targeting therapeutics. STING pathway activation links cytosolic DNA sensing to the induction of type I interferons and pro-inflammatory cytokines, driving innate and adaptive antitumor immunity. Exosomes, mtDNA, micronuclei, and other DAMPs activate cGAS to produce cGAMP, which engages STING and triggers ER-to-Golgi signaling. Pharmacologic STING agonists—including CDN analogs, non-CDN small molecules, and ADC-based payloads—target distinct points along this cascade to amplify immune activation and tumor rejection. (i) Cyclic dinucleotide (CDN) STING agonists: CDN analogs (e.g., ADU-S100, 2′3′-cGAMP analogues, MK-1454, GSK-532, and SB-11285) mimic natural cGAMP and directly engage the STING ligand-binding domain on the ER to initiate downstream signaling. (ii) Non-CDN small-molecule STING agonists: non-CDN agonists (e.g., MSA-1, MSA-2, GSK-3745417, MK-2118, SR-717, TAK-676, and XNS-281) stabilize the active conformation of STING—often independent of cGAS—and exhibit improved cellular penetration relative to CDNs. (iii) Antibody–drug conjugate (ADC) STING agonists: ADC-based STING agonists (e.g., CRD-5500, TAK-500, and XMT-2056) deliver STING-activating payloads selectively to tumor or immune cells through tumor-specific surface antigens, enabling targeted intratumoral STING activation while minimizing systemic toxicity. (iv) Integrated antitumor effects: Collectively, these therapeutic classes converge on the cGAS–STING axis to enhance tumor-intrinsic signaling, innate immune activation, and cytotoxic T cell-mediated tumor rejection. IRF3, interferon regulatory factor-3; GTP, guanosine triphosphate; cGAS, cyclic GMP-AMP synthase; ATP, adenosine triphosphate; STING, stimulator of interferon genes; cGAMP, cyclin guanosine monophosphate–adenosine monophosphate; DAMPs, danger-associated molecular patterns; NF-κB, nuclear factor kappa-light chain-enhancer of activated B cells; mtDNA, mitochondrial DNA, inhibitor of kappa B; CDNs, cyclic dinucleotides; TBK, TANK-binding kinase.

Another CDN agonist, MK-1454 (ulevostinag), was developed by Merck and tested in other solid tumors and lymphomas (NCT03010176). MK-1454 was also administered intratumorally. While the trial was completed, the final results showed that the STING agonist did not improve single-agent efficacy unless combined with pembrolizumab, in which 24% of 25 patients (6/25) achieved partial response, with a 48% disease control rate. In another study of MK-1454 in combination with pembrolizumab for recurrent or unresectable HNSCC, 50% (4/8) of patients achieved a complete or partial response compared to 1/10 in the monotherapy arm (NCT04220866) (256). Mechanistically, both ADU-S100 (MIW815) and MK-1454 (ulevostinag) are synthetic CDNs designed to mimic cGAMP, bind STING directly, and activate the same pathway, bypassing the need for native cGAMP production (as described in **Figure 4**). However, given the limitations of delivery and the suboptimal efficacy of CDNs, the field has moved toward newer generations of STING agonists with improved systemic delivery potential. These drugs include SNX281 and GSK3745417, among others.

### 8.2 Non-CDN small-molecule STING agonists

The second class comprises non-CDN small-molecule STING agonists that aim to improve pharmacokinetics and enable systemic delivery. SNX281, for instance, is a synthetic small molecule that can be administered intravenously (NCT04609579). The trial has now been terminated due to limited single-arm efficacy (257). Similarly, GSK3745417, developed by GlaxoSmithKline, is being evaluated intravenously in relapsed or refractory solid tumors (NCT03843359). This trial is still in dose escalation, with a primary completion date projected for 2025. These efforts represent a promising evolution toward overcoming the constraints of intratumoral dosing and expanding the clinical applicability of STING agonists. Mechanistically, GSK3745417 directly binds STING on the ER, activating TBK1, IRF3, and NF-κB signaling pathways, thereby promoting IFN-I and antitumor immunity (**Figure 4**).

Conceptually, these non-CDN STING agonists (SNX281 and GSK3745417) are designed to bind STING in the same place as cGAMP but with improved pharmacokinetics, enabling systemic delivery rather than local injections. To address many of these challenges, several other innovative strategies to deliver STING agonism into tumors are under exploration, as follows.

#### 8.2.1 Rational evolution from CDNs to non-CDN small molecules

##### 8.2.1.1 SNX281

SNX281 is a small-molecule, non-nucleotide STING agonist designed to overcome the pharmacologic limitations of earlier CDNs. Unlike MK-1454 and ADU-S100, which required intratumoral injection, SNX281 is administered intravenously, allowing for systemic delivery and improved tumor accessibility. This design supports the activation of STING signaling across both primary and metastatic lesions, broadening therapeutic reach beyond injection-accessible tumors. Ongoing trial (NCT04609579) evaluating its safety and preliminary efficacy in advanced solid tumors and lymphomas was terminated due to the sponsor’s reasons.

##### 8.2.1.2 GSK3745417

GSK3745417 represents another generation of systemically deliverable STING agonists with distinct pharmacokinetic advantages over nucleotide-based predecessors. This non-cyclic dinucleotide small molecule is administered intravenously, ensuring wide distribution across tumor sites and consistent systemic exposure. Current early-phase clinical trials (NCT03843359) are assessing tolerability and dosing in patients with relapsed or refractory solid tumors. The design prioritizes systemic immune activation while reducing the risk of the local-only effect observed with intratumoral STING agonists.

#### 8.2.2 Comparative perspective

Together, SNX281 and GSK3745417 illustrate the rational evolution of STING-targeted therapeutics. By addressing the limitations of CDNs, namely, short half-life, poor systemic distribution, and the requirement for intratumoral injection, these small molecules expand the scope of STING activation into systemically disseminated disease. Thus, compared with older CDNs (intratumoral only, short half-life, and poor systemic distribution), SNX281 and GSK3745417 represent new-generation systemically deliverable small-molecule STING agonists that offer broader tumor access and clinical versatility.

### 8.3 Other STING agonists

#### 8.3.1 Immune-stimulating antibody-conjugate modalities

These are antibodies targeting tumor-associated antigens, conjugated to STING agonist payloads to activate STING within the tumor or TME selectively. ADCs are being engineered to deliver STING agonists selectively to tumor cells. XMT-2056, for example, is an ADC targeting HER2-positive tumors and is currently undergoing a Phase 1 trial in advanced HER2^+^ solid tumors (NCT05514717). Studies have also demonstrated “tumor cell-directed STINGa ADCs” that activate STING in both tumor and myeloid cells when systemically administered, with improved safety and reduced systemic cytokine release relative to free agonists (258). The other form is TAK-500. TAK-500 is described as an immune-stimulating antibody conjugate (ISAC): an anti-CCR2 antibody linked with a STING agonist base (related to TAK-676) *via* a protease-cleavable linker (11). It is being studied with or without pembrolizumab in advanced solid tumors to overcome checkpoint inhibitor resistance and reprogram tumor-associated macrophage or myeloid populations. Alternative approaches include using engineered bacteria or exosomes to release STING agonists *in situ* within the TME, improving both the precision and potency of delivery (259, 260).

#### 8.3.2 Exosome-mediated STING agonist delivery

The STING pathway plays a central role in innate immunity and is increasingly recognized as a therapeutic target in oncology. The activation of STING within APCs induces type I interferon release and cytokine-driven antitumor immunity. However, barring an administration bottleneck, the clinical translation of CDNs and their derivatives has been constrained by poor pharmacokinetics, limited biostability, rapid systemic clearance, and inefficient cytosolic delivery, all of which hinder effective tissue accumulation and therapeutic potency (260).

To address these limitations, exosomes have emerged as promising carriers due to their intrinsic cellular uptake capacity and biocompatibility. A novel strategy, termed iExoSTINGa, leverages engineered exosomes to deliver the STING agonist cGAMP directly to APCs (259). In this study, McAndrews and coworkers demonstrated that iExoSTINGa enhanced dendritic cell uptake of the agonist, leading to increased CD8^+^ T-cell activation and the superior suppression of B16F10 melanoma growth compared with free STING agonist (259). These findings underscore the therapeutic potential of exosome-mediated delivery platforms in augmenting STING-targeted cancer immunotherapy.

#### 8.3.3 Engineered bacterial strains producing STING agonists

This is a novel method that uses live bacteria modified to produce STING agonists within the TME. These approaches may exploit hypoxic conditions or tumor-specific promoters to restrict agonist production to the lesion site. A notable example is SYNB1891, an engineered *Escherichia coli* Nissle strain, which is administered intratumorally either alone or in combination with the immune checkpoint inhibitor atezolizumab in patients with advanced malignancies (261). According to the clinical trial, treatment with SYNB1891 resulted in clear evidence of STING pathway activation and target engagement, demonstrated by the upregulation of interferon-stimulated genes, chemokines/cytokines, and T-cell response genes in core biopsies collected before dosing and 7 days after the third weekly injection. A dose-related increase in serum cytokines was also observed, along with stable disease in four participants who had previously been refractory to PD-1/PD-L1 blockade (NCT04167137). Overall, repeat intratumoral injections of SYNB1891, both as monotherapy and in combination with atezolizumab, were safe, well-tolerated, and biologically active, supporting its potential as a novel immunotherapy strategy with strong evidence of STING pathway engagement, including the upregulation of interferon-stimulated genes.

Apart from the above-listed categories, CRD3874-SI (also called ONM-501) is also among other newer candidates that represent a dual STING agonist with both TLR and STING pathway engagement, currently under evaluation in a first-in-human Phase I study in advanced solid tumors and lymphomas (NCT06022029). Administered intratumorally, ONM-501 is also being tested in combination with the anti-PD-1 agent cemiplimab. In addition, CRD3874-SI is also undergoing a phase 1 trial at Memorial Sloan Kettering (MSK) against sarcoma and Merkel cell carcinoma (NCT06021626). This dual strategy aims to enhance cross-priming and immune infiltration, capitalizing on the synergy between innate sensing and checkpoint blockade.

Taken together, these strategies exemplify the multifaceted nature of STING agonist development, spanning direct agonists, conjugates, and biological delivery systems. While initial trials with CDNs underscored delivery and efficacy limitations, newer formulations and combination strategies are reinvigorating the clinical promise of this pathway. As previously iterated, the central dogma of cGAS–STING activation suggests that tumor-derived cytosolic DNA triggers cGAS to generate cGAMP, which activates STING in the endoplasmic reticulum. This leads to phosphorylation of TBK1 and IRF3, type I interferon production, and NF-κB activation. Intrinsic to this pathway is the induction of tumor cell senescence and growth arrest; extrinsic effects include activating antigen-presenting cells, enhancing T-cell recruitment via CXCL9/10, and promoting NK cell-mediated cytotoxicity.

## 9 Clinical development of STING agonists

Several STING agonists have advanced to the early-phase clinical evaluation, primarily as intratumoral agents designed to convert immunologically “cold” tumors into inflamed, T cell-permissive microenvironments. *ADU-100* (MIW815) (NCT02675439), a synthetic CDN STING agonist, is the first to be evaluated in phase I trials as a monotherapy and in combination with pembrolizumab, spartalizumab, or ipilimumab for advanced solid tumors and lymphomas. While intratumoral administration was generally well-tolerated, the objective response rate (ORR) was modest, with clinical benefit limited to injected lesions and transient immune activation, highlighting the challenges in achieving durable systemic antitumor immunity (252, 255). Similarly, *MK-1454* (NCT03010176), developed by Merck, demonstrated limited single-agent activity in early-phase studies but showed improved immune activation when combined with pembrolizumab, albeit with low overall response rates (ORRs). Across trials, common toxicities include local injection-site reactions, flu-like symptoms, and cytokine-associated adverse events, consistent with on-target innate immune activation (6).

*SB11285* is a systemically administered (intravenous) CDN agonist being evaluated in a Phase 1/1b dose-escalation and expansion study as both a monotherapy and in combination with atezolizumab in patients with advanced solid tumors (NCT04096638). Although no public response data have been reported to date, the primary endpoints of the study are safety and tolerability, as well as assessments of pharmacokinetics and pharmacodynamics (PK/PD). Others tested in a human Phase 1 trial as monotherapy or in combination with anti-PD1 include *BI 1703880* (NCT05471856) and *GSK3745417* (NCT03843359), both non-CDN STING agonists. The majority of the STING agonists remain in early-phase clinical development, reflecting an ongoing effort to define safe and biologically relevant efficacy rather than a lack of translational potential. **Table 2** lists the ongoing STING agonists currently being evaluated. These early clinical experiences underscore the translational gap between robust preclinical STING activation and modest clinical efficacy, emphasizing the importance of optimizing dosing schedules, route of administration, and rational combination strategies to avoid immune exhaustion or chronic inflammatory signaling.

While several STING agonist trials remain formally listed as “ongoing”, most completed studies to date demonstrated limited clinical efficacy, leading to early termination or stalled development of multiple candidates. Collectively, this highlights a fundamental disconnect between innate immune pathway activation and sustained antitumor immunity. These findings suggest that the main obstacle in STING agonist development is not the accessibility of the pathway but rather the challenge of achieving precisely timed and localized activation that minimizes chronic inflammatory responses and prevents immune exhaustion. The subsequent section discusses these challenges in detail, including the biological complexities involved, tumor heterogeneity, and potential strategies to overcome these obstacles.

## 10 Clinical challenges and future opportunities for cGAS–STING agonists in cancer therapy

Despite the considerable promise of cGAS–STING agonists in preclinical cancer models, their clinical translation faces significant obstacles. These challenges span both the biological complexity of the cGAS–STING pathway and the practical hurdles of therapeutic application. Notably, clinical trials of STING agonists have been characterized by immune-related toxicities, tumor-intrinsic resistance mechanisms, delivery inefficiencies, and patient variability (8), underscoring the need for carefully tailored strategies in the design, development, and deployment of STING-based therapies. The most significant is the context-dependent duality of sting signaling, in which acute activation promotes antitumor immunity, whereas chronic or excessive activation drives the NF-κB-IL-6-STAT3-mediated expansion of MDSCs and Forkhead box P3 (FOXP3)+ regulatory T cells, leading to immune suppression in tumors such as HNSCC, TNBC, and tongue squamous cell carcinoma (TSCC). Tumor-intrinsic resistance arising from a low or epigenetically silenced cGAS–STING pathway (as noted in GBM and melanoma) further limits responsiveness, while oxidative and metabolic stress within the TME can impair STING activation through redox-mediated modifications.

In parallel, direct immune cell toxicity, including T-cell dysfunction, induced by ER stress and calcium imbalance, further narrows the therapeutic window of STING agonists. These challenges are compounded by TME heterogeneity, the adaptive upregulation of inhibitory pathways like PD-L1 and IDO1, and fundamental structural differences between murine and human STINGs that undermine translational predictability. Collectively, these factors (highlighted in **Table 3**) help explain the modest clinical efficacy observed and underscore the need for refined dosing strategies, biomarker-driven patient selection, rational combination approaches, and human-relevant model systems in the development of next-generation STING-based immunotherapies.

**Table 3:** Current challenges and solutions for cGAS–STING-targeted therapies in clinical applications.

Category: biological complexities	Challenge	Tumor type	Potential solutions
Dual role of STING signaling	Acute activation promotes antitumor immunity, but chronic activation can drive MDSC and Foxp3+ Treg expansion and NF-κB–IL-6–STAT3 signaling.	HNSCC (92), TNBC (269), TSCC (270)	Optimize dosing and duration to avoid chronic activation; combine with IL-6/STAT3 inhibitors
Tumor-type specific responses	Some tumors are intrinsically resistant due to lack of cGAS/STING expression or epigenetic silencing	GMB (151), melanoma (97)	Use epigenetic modulators (e.g., DNMT or HDAC inhibitors); select patients based on STING expression
Redox state and metabolic interference	ROS or ferroptosis-associated carbonylation inhibits STING palmitoylation/activation		Antioxidant therapy or GPX4 modulation to preserve STING function in oxidative environments
Immune cell toxicity	STING activation can lead to T-cell death via ER stress and calcium imbalance		Use lower doses or modified STING agonists; co-target ER stress pathways
Heterogeneity of TME	STING activation may recruit suppressive myeloid cells or activate T cells, depending on context		Personalize therapy based on TME profiling; combine with MDSC or TAM-depleting agents
Crosstalk with other pathways	STING activation upregulates PD-L1, IDO1, potentially limiting response	TSCC, mouse melanoma, OSCC (271), TNBC (269)	Combine with immune checkpoint inhibitors or IDO1 inhibitors
Species-specific differences	Mouse and human STING differ structurally and functionally, limiting translational accuracy		Use humanized mouse models or organoid systems; develop human-specific STING agonists
Category: Clinical application hurdles	Challenge	Potential solutions	
Delivery challenges	CDNs are hydrophilic, rapidly degraded, and poorly taken up by cells	Use nanoparticle, liposome, or polymer-based delivery systems; develop non-CDN small-molecule agonists	
Systemic toxicity and off-target effects	STING is widely expressed; systemic activation can trigger inflammation or autoimmunity (261)	1. Intratumoral delivery 2. Develop tissue-specific agonists, such as STING agonists conjugated with antibodies, which can be specifically delivered to sites expressing tumor-specific antigens 3. Conditional activation systems 4. Identify biomarkers to enable patient selection	
Lack of predictive biomarkers	Absence of robust biomarkers for response or resistance	Identify and validate predictive biomarkers (e.g., STING levels, TILs, and cGAMP export/import genes)	
Dose optimization and therapeutic window	Narrow therapeutic window with variable efficacy	Use adaptive dosing regimens; refine intratumoral vs systemic delivery strategies	
Tumor burden and disease stage	Late-stage tumors with suppressive TME resist STING therapy	Combine with debulking regimens (e.g., surgery and radiation); use in adjuvant or early-stage settings	
Combination strategies still evolving	Optimal therapeutic combinations and timing remain uncertain	Conduct preclinical screens and rational trials combining with checkpoint inhibitors, radiation, and chemotherapy	
Pharmacokinetics and bioavailability	CDNs have poor tumor retention and are degraded by ENPP1	Use ENPP1 inhibitors concurrently; develop STING agonist prodrugs or long-acting delivery platforms	

MDSCs, myeloid-derived suppressor cells; FOXP3, Forkhead box p3; Tregs, regulatory T cells; NF-κB, nuclear factor kappa-light chain-enhancer of activated B cells; IL-6, interleukin-6; STAT3, signal transducer and activator of transcription 3; TNBC, triple-negative breast cancer; HNSCC, head and neck squamous cell carcinoma; DNMT, DNA methyltransferase; HDAC, histone deacetylase; DPX4, glutathione peroxidase 4; ER, estrogen receptor; TAM, tumor-associated macrophage; OSCC, oral squamous cell carcinoma; IDO1, indoleamine dioxygenase-1; PD-L1, programmed-death ligand-1; CDNs, cyclic dinucleotides; TILs, tumor-infiltrating lymphocytes; cGAMP, cyclic guanosine monophosphate-adenosine monophosphate; TME, tumor microenvironment; ENPP1, ectonucleotide pyrophosphatase/phosphodiesterase-1; TSCC, tongue squamous cell carcinoma.

## 11 Conclusion

Collectively, the available evidence underscores the dualistic nature of cGAS–STING signaling, in which precise modulation determines whether tumors mount productive immune activation or shift toward chronic inflammation and immune escape. Understanding the determinants of pathway silencing, the molecular thresholds that distinguish acute from chronic activation, and the TME microenvironmental cues governing therapeutic efficacy will be critical for future progress. Advancing biomarker-guided deployment of STING-targeted therapies, particularly in rational combinations, offers a promising path toward durable clinical benefit.
